# Familial Loss of a Loved One and Biological Aging: NIMHD Social Epigenomics Program

**DOI:** 10.1001/jamanetworkopen.2024.21869

**Published:** 2024-07-29

**Authors:** Allison E. Aiello, Aura Ankita Mishra, Chantel L. Martin, Brandt Levitt, Lauren Gaydosh, Daniel W. Belsky, Robert A. Hummer, Debra J. Umberson, Kathleen Mullan Harris

**Affiliations:** 1 Department of Epidemiology, Mailman School of Public Health, Columbia University, New York, New York; 2 Robert N. Butler Columbia Aging Center, Mailman School of Public Health, Columbia University, New York, New York; 3 Department of Psychology, College of Humanities and Social Sciences, North Carolina State University, Raleigh; 4 Carolina Population Center, University of North Carolina at Chapel Hill; 5 Department of Epidemiology, Gillings School of Global Public Health, University of North Carolina at Chapel Hill; 6 Department of Sociology, University of Texas at Austin; 7 Department of Sociology, University of North Carolina at Chapel Hill

## Abstract

**Importance:**

The link between familial loss of a loved one and long-term health decline is complex and not fully understood.

**Objective:**

To test associations of losing a parent, sibling, child, or partner or spouse with accelerated biological aging.

**Design, Setting, and Participants:**

Data from the National Longitudinal Study of Adolescent to Adult Health, a US population-based longitudinal cohort study, were analyzed. Participants were enrolled from 1994 to 1995 for wave 1, while in grades 7 to 12, and followed up through wave 5 in 2018. The study analyzed participant reports of loss collected at each wave from 1 to 5 over 24 years and used a banked wave 5 blood sample for subsequent DNA methylation testing and epigenetic clock calculation from 2018 to 2024. Data were analyzed from January 2022 to July 2024.

**Exposure:**

Loss of biological parents or parental figures, partners or spouses, siblings, or children at waves 1 to 3 or during childhood, adolescence (aged <18 years), or adulthood at wave 4 to wave 5 (aged 18-43 years).

**Main Outcomes and Measures:**

Biological aging assessed from blood DNA methylation using the Horvath, PhenoAge, GrimAge, and DunedinPACE epigenetic clocks at wave 5.

**Results:**

Data from 3963 participants were analyzed, with a weighted mean (range) age of 38.36 (36.78-39.78) years at wave 5; 2370 (50.3%) were male, 720 (15.97%) were Black, 400 (8.18%) were Hispanic, and 2642 (72.53%) were White. Nearly 40% of participants experienced loss by wave 5 when they were aged 33 to 43 years, and participants who were Black (379 participants [56.67%]), Hispanic (152 participants [41.38%]), and American Indian (18 participants [56.08%]) experienced a greater proportion of losses compared with White participants (884 participants [34.09%]). Those who experienced 2 or more losses tended to have older biological ages for several of the clocks (PhenoAge β = 0.15; 95% CI, 0.02 to 0.28; GrimAge β = 0.27; 95% CI, 0.09 to 0.45; DunedinPACE β = 0.22; 95% CI, 0.10 to 0.34) compared with those with no losses. In contrast, there were no associations with 2 or more losses for the Horvath clock (β = −0.08; 95% CI, −0.23 to 0.06).

**Conclusions and Relevance:**

This study reveals associations between various measures of loss experienced from childhood to adulthood and biological aging in a diverse sample of the US population. These findings underscore the potentially enduring impact of loss on biological aging even before middle age and may contribute to understanding racial and ethnic disparities in health and mortality.

## Key Points

**Question:**

Is the experience of losing a loved one associated with accelerated biological aging?

**Findings:**

In a cohort study of 3963 participants from the National Longitudinal Study of Adolescent to Adult Health, nearly 40% experienced the loss of a close relation by adulthood. Participants who had experienced a greater number of losses exhibited significantly older biological ages compared with those who had not experienced such losses.

**Meaning:**

These findings suggest that loss can accelerate biological aging even before midlife and that frequency of losses may compound this, potentially leading to earlier chronic diseases and mortality.

## Teaser

This cohort study evaluates associations between losing a loved one and accelerated biological aging.

## Introduction

The relationship between bereavement and health across the life course is well-established and enduring.^1^ However, there are indications that certain life stages may be more susceptible to the health and mortality risks associated with loss. For instance, the loss of a parent or sibling in early life is notably traumatic and linked to poor mental health, cognitive impairment, increased cardiometabolic risk, and higher mortality risk in later life.^2,3,4,5^ Nonetheless, the death of a close family member at any age poses health risks, such as heightened cardiovascular, mortality, and dementia risks in adulthood.^1,6,7,8^ Repeated family losses over one’s life further compound these health risks.^6^ The impacts of loss may persist or become apparent long after the event.^5,6,8,9^

The mechanisms connecting loss to poor health and mortality remain unclear. There are at least 2 pathways, each with intermediate processes. One pathway begins with deficits in material and social resources formerly provided by the person who dies. Another begins with the psychological distress of bereavement. Both can ultimately result in dysregulation of biological systems due to changes in health behaviors related to bereavement-related distress and stress from economic constraints. One framework that integrates the impacts of these diverse pathways and intermediate processes on human health is emerging research on biological aging.^10^

Biological aging refers to the progressive loss of integrity and resilience capacity in our cells, tissues, and organs as we grow older.^11^ While there are no universally accepted methods to measure biological aging in humans, the best-validated of these methods are a family of DNA-methylation algorithms known as epigenetic clocks, including several that show robust evidence for prediction of future morbidity and mortality.^10,12,13^ However, few studies have empirically explored the association between loss across developmental periods and DNA methylation markers of aging, and rarely have these investigations been conducted in racially and ethnically diverse population-based studies.

To address existing gaps, we investigated if familial loss and quantity of loss, occurring either in early life or adulthood, were associated with biological aging in a diverse, population-based sample. Acknowledging prior research on racial disparities in loss experiences and variability in exposure histories,^6,14^ we also examined the interaction between loss and race on biological aging. We analyzed survey responses from waves 1 to 5 of the National Longitudinal Study of Adolescent to Adult Health (Add Health), coupled with new epigenetic data from wave 5.

## Methods

We used data from wave 1 to wave 5 of Add Health, which has been previously described^15^ and is a US nationally representative cohort, following up participants since the 1994 to 1995 school year. Wave 5 took place between 2016 and 2018 and completed interviews with 12 300 participants. During wave 5, participants were invited for an additional home examination where a venous blood sample was gathered. Of the 7995 individuals who agreed to the home examination, 5381 were successfully visited, and 4940 provided a blood sample. We used data from 3963 participants who had a blood sample, reported losses at each wave, and reported data on covariates. We used weighting to ensure representation of the Add Health cohort since wave 1.^15^ The weights account for the original sampling design, attrition to the current wave 5, and differential consent to the in-home blood collection.^16^

Participants and their parents or caregiver in childhood provided written consent at waves 1 through 2; at age 18 years, only the participant’s consent was obtained (waves 3-5). The study was approved by the institutional review boards of Columbia University and the University of North Carolina, Chapel Hill. We followed the Strengthening the Reporting of Observational Studies in Epidemiology (STROBE) reporting guidelines.^17^

### Epigenetic Clocks

From 2018 to 2024, we conducted epigenome-wide profiling and construction of clocks, using whole-blood DNA for 4700 participants at wave 5 using the Infinium MethylationEPIC BeadChip array (Illumina, Inc), as previously described.^18^ After standard quality control, we calculated 4 biological clocks, including GrimAge, PhenoAge, and Horvath, to assess epigenetic age acceleration,^19,20,21,22^ and the DunedinPACE to assess pace of biological aging.^23,24^ For analysis, Horvath, GrimAge, and PhenoAge were first residualized on chronological age and residuals were *z*-transformed to allow comparability across measures. DunedinPACE is a measure of the pace of aging—how rapidly or slowly a person is aging.^24^ It is a rate, not an age, and so no residualization was conducted, but values were *z*-transformed for comparability.

### Familial Loss

Familial loss and the timing of loss variables were derived from waves 1 to 5. We included the following from each wave: (1) parental death (biological and parent figures), (2) death of a sibling, (3) death of a spouse or partner, and (4) death of a child. We calculated the total number of losses by pooling across 5 waves, using only new reports of loss. Number of losses were coded as 0, 1, or 2 or more losses.

#### Parental Loss Across Waves 1 to 5

Given research suggesting a strong association between parental loss, in particular, and the health of surviving children,^25,26,27^ in addition to the fact that it was the most common type of loss in Add Health, we assessed parental loss at any time point across waves 1 to 5. The parental loss variable included the death of parents and/or parent figures at any wave.

#### Developmental Period of Loss

To classify loss at varying developmental periods, we used survey reports of parental and sibling loss reported on surveys from wave 1 to 2 by identifying whether the participant was aged less than 18 years at the time of the loss. To classify loss among participants aged less than 18 years, we used the month and year of parental and sibling deaths reported at wave 3 or 4 or as reported in the last 12 months on wave 5. We also used reports of death of a partner or spouse and child from waves 3 to 5.

We developed variables for any loss in childhood or adolescence and any loss in adulthood based on reported loss timing. Any loss in childhood or adolescence includes deaths of parents (biological and parental figures) and siblings before age 18 years. Any loss in adulthood encompasses deaths of parents, siblings, spouses or partners, and children when the participant was 18 or older. Additionally, we created variables for parental loss in childhood or adolescence (death of a parent or parental figure before age 18 years) and parental loss in adulthood (death of a parent or parental figure at age 18 years or older).

### Covariates

Several covariates were included in the analytic models based on known or potential confounders and directed acyclic graphs.^10,13,28,29,30^ Chronological age at blood draw was included as a continuous variable. Participant self-identified race and ethnicity was based on the race or ethnicity that the participant indicated that they most strongly identified with at the wave 5 survey, including Asian, Black, Hispanic, American Indian, other, Pacific Islander, or White. Participants that responded as other did not identify with any race or ethnic categories or identified as multiracial only. In a small proportion of cases (9 participants) where wave 5 information was missing or unavailable, wave 1 was used to identify self-reported race and ethnicity. We present loss for all racial and ethnic groups where sample sizes permit. In our regression models, we categorized race as Black, Hispanic, and a combined category for all other racial groups due to limited sample sizes. Respondent’s report on sex assigned at birth at wave 5 was used to categorize participants as female or male. To account for environmental or neighborhood influences on epigenetic aging in early life, we included a variable that represented the proportion of households with an income below the poverty line in 1989 based on participant addresses at wave 1. We also adjusted for the number of household members in wave 1, to account for the increased likelihood of loss among those with greater members. We also included parental educational attainment based on the highest attainment of either parent at wave 1 (high school degree or less, some college, and college or higher). Smoking was based on parent’s self-report of smoking at wave 1. We adjusted for epigenetic assay batch (batch 1, 41.34%; batch 2, 56.51%; batch 3, 1.15%). We also conducted sensitivity analyses by adjusting for cell counts as described in eTable 1, eTable 2, eTable 3, and eTable 4, and for duration since loss in eTable 5, eTable 6, eTable 7, and eTable 8 in Supplement 1.

### Statistical Analysis

We used survey linear regression models for assessing the associations between each number of losses and each of the biological aging measures, adjusting for all covariates described previously. Models were estimated and graphs created using the survey,^31,32,33,34^ jtools,^35^ and ggplot2^36,37^ packages in R version 4.2.1 (R Project for Statistical Analysis).^35,36,37,38,39^

In models investigating timing of loss (any loss or parental loss), we included a variable indicating loss during childhood or adolescence and a variable indicating loss during adulthood so that we could assess the association of childhood or adolescence and adulthood loss independently of one another. Following Add Health guidelines,^16,38^ analyses accounted for complex sampling features (ie, probability weights, nesting of individuals in primary sampling units, no siblings, and stratification) so that results represent the population of individuals who were enrolled in grades 7 to 12 in the US in 1994 to 1995.^16^ We used 95% CIs to evaluate statistical significance.

Racial and ethnic disparities exist in the frequency and nature of losing loved ones, often due to racism and a history of disadvantage.^3,6,40,41,42,43^ Race is not a biological factor and while race itself is not racism, it is a sociopolitical and contextual construct linked to greater exposure to adverse environments and health disadvantages from a history of oppression.^44,45,46,47,48^ Furthermore, clocks, mainly developed in predominantly White samples, show varying sensitivities to social and environmental exposures across different races.^49^ Thus, we presented exposure to loss by race and included an interaction term between loss and race in our biological aging models to explore effect modification by race.^44,47,48^ Interaction analyses focused on Black (and White participants due to the smaller number of other racial and ethnic categories) (see Table). We applied the same models as in the overall sample, adding an interaction term between loss and race, considering interaction terms significant at a 2-sided *P* < .10. Data were analyzed from Janaury 2022 to July 2024.

**Table.** Population Descriptive Statistics (Weighted Proportions and Unweighted Ns)

Characteristic	Participants (N = 3963), No. (weighted %)
Age at wave 5, median (weighted IQR)	38.36 (36.78-39.78)
Sex assigned at birth	
Female	2370 (49.97)
Male	1593 (50.03)
Race and ethnicity	
Asian	145 (1.89)
Black	720 (15.97)
Hispanic	400 (8.18)
American Indian^a^	28 (0.89)
Other^a^^,^^b^	11 (0.34)
Pacific Islander^b^	17 (0.21)
White	2642 (72.53)
Parental education, wave 1	
High school	1261 (35.15)
Some college	1196 (30.72)
College or higher	1506 (34.14)
No. of household members, wave 1, median (weighted IQR)	3.00 (2.00-4.00)
Proportion of households under poverty, wave 1, mean (SE)	0.14 (0.01)
Caregiver smoking, wave 1	1034 (27.94)
Any loss, waves 1-5	1491 (38.49)
No. of losses, waves 1-5	
1	1066 (28.84)
≥2	425 (9.65)
Types of loss, wave 1-5	
Any loss in childhood	351 (7.97)
Any loss in adulthood	1095 (28.59)
Parental loss	1245 (32.37)
Parental loss in childhood	263 (6.31)
Parental loss in adulthood	1019 (26.73)
Any loss by sex	
Female	911 (39.12)
Male	580 (37.87)
Parental loss by sex	
Female	755 (32.81)
Male	490 (31.92)
Any loss by race and ethnicity	
Asian	41 (28.83)
Black	379 (56.67)
Hispanic	152 (41.38)
American Indian^b^	18 (56.08)
Other^c^	NA
Pacific Islander^c^	NA
White	884 (34.09)
Parental loss by race and ethnicity	
Asian	34 (25.03)
Black	323 (48.72)
Hispanic	127 (31.70)
American Indian^b^	14 (41.08)
Other^c^	NA
Pacific Islander^c^	NA
White	732 (28.78)
Biological aging clock raw scores, wave 5, mean (SE)	
Horvath	39.16 (0.17)
PhenoAge	30.02 (0.19
GrimAge	52.50 (0.17)
DunedinPACE	0.99 (0.00)
Biological aging clock residuals, wave 5, mean (SE)	
Horvath	0.14 (0.09)
PhenoAge	0.07 (0.13)
GrimAge	0.31 (0.14)
Biological aging clock *z* scores, wave 5, mean (SE)	
Horvath	0.03 (0.02)
PhenoAge	0.01 (0.02)
GrimAge	0.08 (0.03)
DunedinPACE	−0.02 (0.04)

Abbreviation: NA, not available.

^a^ Estimate based on small sample size (eg, <30).

^b^ Participants who self-identified as other did not identify with any other race or ethnic category or indicated multiracial only.

^c^ Estimate cannot be presented because of deductive disclosure risks based on Add Health restricted data use policy.

## Results

The analytical sample included 3963 Add Health participants who had data on all variables in the analysis (Table). The weighted sample mean (range) age was 38.36 (36.78-39.78) years at wave 5; 2370 (50.3%) were male, 720 (15.97%) were Black, 400 (8.18%) were Hispanic, and 2642 (72.53%) were White. Almost 40% of participants experienced 1 or more losses (Table). Most losses occurred in adulthood and parental loss was more common in adulthood vs in childhood or adolescence (26.73% vs 6.31%) (Table). Loss was most frequently reported by Black (379 participants [56.67%]), and American Indian (18 participants [56.08%]) participants, followed by Hispanic (152 participants [41.38%]), White (884 participants [34.09%]), and Asian (41 participants [28.83%]) participants.

### Number of Losses

**Figure 1.**
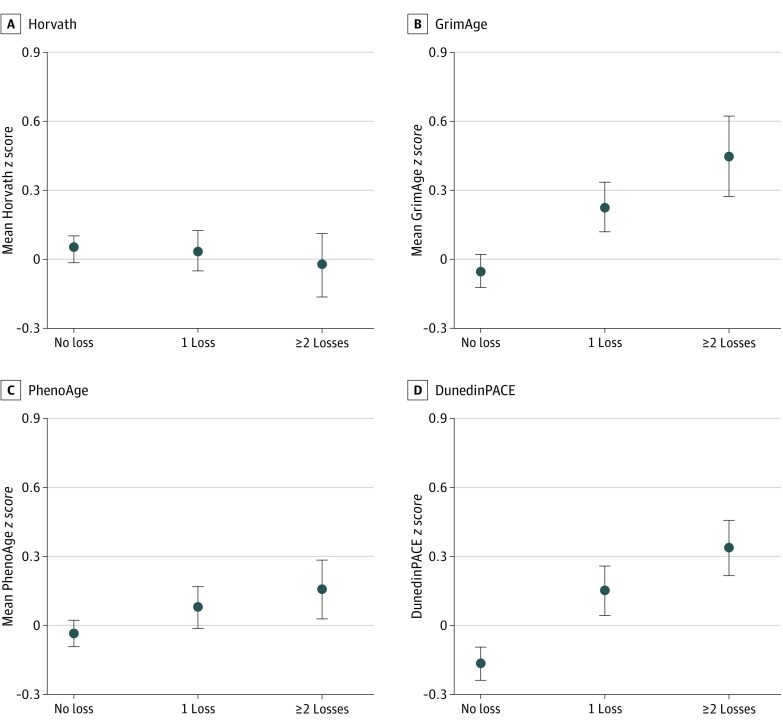
Weighted Means for Biological Aging Clocks (*z* Scored) by Number of Losses Dots indicate means and bars indicate SEs. Values are weighted means.

The mean *z* score and SEs for each biological clock by number of losses are presented in Figure 1. As the number of losses increased, GrimAge, PhenoAge, and DunedinPACE increased. There were no discernible differences in the Horvath clock by number of losses (β = −0.08; 95% CI, −0.23 to 0.06).

**Figure 2.**
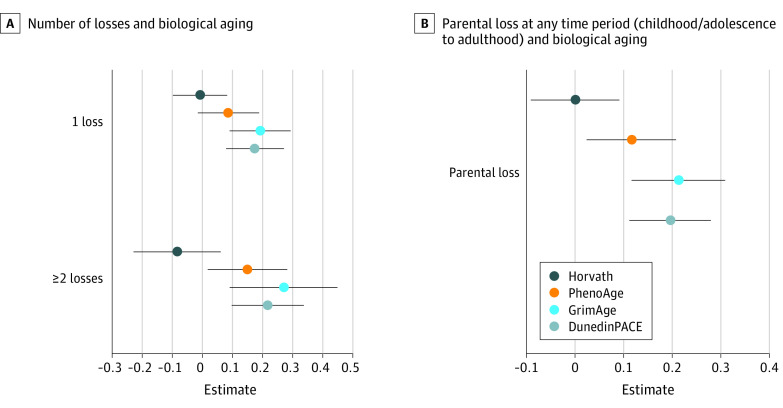
Number of Losses and Biological Aging and Parental Loss at Any Time Period (Childhood/Adolescence to Adulthood) and Biological Aging Clock values are *z* scored. Models adjusted for age, race and ethnicity, parental education, gender, proportion of households in poverty, number of household members, caregiver smoking, and epigenetic assay batch. See eTable 1 in Supplement 1 for full model specifications and estimates.

In adjusted models, participants who experienced only 1 loss vs no loss had older biological ages for GrimAge (β = 0.19; 95% CI, 0.09 to 0.29) and DunedinPACE (β = 0.17; 95% CI, 0.08 to 0.27), but not for PhenoAge or Horvath clocks (See Figure 2A; eTable 9 in Supplement 1). Participants with 2 or more losses tended to have significantly older biological ages for several of the biological clocks (PhenoAge β = 0.15; 95% CI, 0.02 to 0.28; GrimAge β = 0.27; 95% CI, 0.09 to 0.45; DunedinPACE β = 0.22; 95% CI, 0.10 to 0.34). Two or more losses, however, were not associated with biological aging as measured by the Horvath clock (β = −0.14; 95% CI, −0.40 to 0.12). All results are shown in eTable 9 in Supplement 1.

### Parental Loss From Childhood/Adolescence to Adulthood

Participants who lost a parent in either childhood or adolescence or adulthood tended to have an older PhenoAge (β = 0.12; 95% CI, 0.02-0.21), GrimAge (β = 0.21; 95% CI, 0.12-0.31), and DunedinPACE (β = 0.19; 95% CI, 0.11-0.28) than participants who had no losses. The Horvath clock was not associated with parental loss. Results are shown in Figure 2B and eTable 10 in Supplement 1.

### Timing of Loss

**Figure 3.**
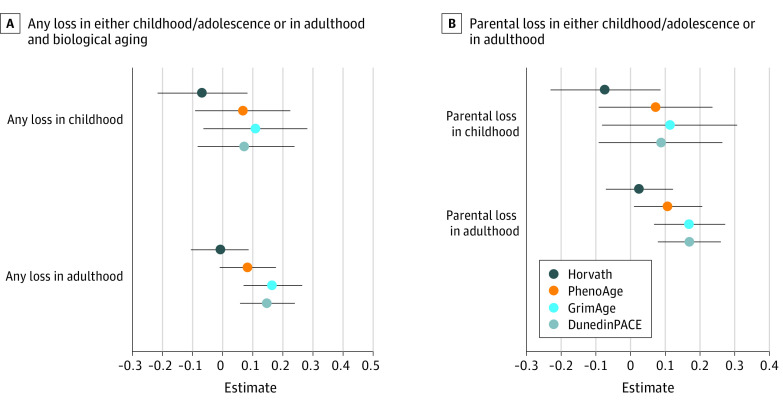
Any Loss in Either Childhood/Adolescence or in Adulthood and Biological Aging and Parental Loss in Either Childhood/Adolescence or in Adulthood and Biological Aging Clock values are *z* scored. Models adjusted for age, race and ethnicity, parental education, gender, proportion of households in poverty, number of household members, caregiver smoking, and epigenetic assay batch. See eTable 1 in Supplement 1 for full model specifications and estimates.

Participants who experienced any losses during childhood or adolescence (adjusting for later life loss) exhibited similar biological aging to those who experienced no losses during childhood (Horvath β = −0.07; 95% CI, −0.22 to 0.08; PhenoAge β = 0.07; 95% CI, −0.09 to 0.23; GrimAge β = 0.11; 95% CI, −0.06 to 0.28; DunedinPACE β = 0.08; 95% CI, −0.08 to 0.24). Results are shown in Figure 3A and eTable 11 in Supplement 1.

In contrast, participants who experienced any loss during adulthood, adjusted for childhood or adolescent losses, exhibited older biological ages as compared with peers with no adulthood loss for GrimAge (β = 0.16; 95% CI, 0.07-0.26) and DunedinPACE (β = 0.15; 95% CI, 0.06-0.24), but not for PhenoAge or Horvath clocks. Results are shown in Figure 3A and eTable 11 in Supplement 1.

Participants who had lost a parent during childhood did not differ in their biological aging from participants who had not, after adjusting for loss of a parent in adulthood (Figure 3B; eTable 12 in Supplement 1). In contrast, participants who lost a parent during adulthood, adjusting for loss of a parent in childhood, exhibited older biological ages for several clocks (PhenoAge β = 0.10; 95% CI, 0.01 to 0.20; GrimAge β = 0.17; 95% CI, 0.07 to 0.27; DunedinPACE β = 0.17; 95% CI, 0.08 to 0.26), but not the Horvath Clock (β = 0.02; 95% CI, −0.07 to 0.12). Results are shown in Figure 3B and eTable 12 in Supplement 1.

### Sensitivity Analyses

Controlling for cell counts was not associated with overall effect sizes (see eTable 1, eTable 2, eTable 3, and eTable 4 in Supplement 1). Similarly, inclusion of time since parental loss showed very little difference in estimates overall (see eTable 5, eTable 6, eTable 7, and eTable 8 in Supplement 1). We also examined the impact of loss by parental gender (maternal versus paternal) and found no significant differences (see eTable 17 in Supplement 1), and there were no significant interactions between loss in childhood and adulthood on epigenetic aging (see eTable 18 and eTable 19 in Supplement 1)

### Interactions With Race

Race by loss interaction model results are reported in eTable 13, eTable 14, eTable 15, and eTable 16 in Supplement 1. There were no statistically significant interactions between race and loss and biological clocks at the α = .10 level.

## Discussion

Our findings suggest that accelerated biological aging may represent a key mechanism associating exposure to death of family members with later life risk of morbidity and mortality. Among US adults aged 33 to 44 years, experiences of loss were consistently associated with older biological age as measured by PhenoAge and GrimAge clocks and faster pace of aging as measured by the DunedinPACE, with a higher burden of loss exhibiting a dose-response association with the extent of accelerated biological aging. In contrast, there was no association between loss and biological aging as measured by the Horvath clock. Losses experienced during adulthood showed greater associations with biological aging as compared with losses experienced during childhood or adolescence. Findings suggest that experiences of loss may contribute to trajectories of biological aging even before the onset of midlife, thus helping to set the course for earlier risk of chronic disease and mortality.

There is an increasing amount of research indicating that prolonged stress and related traumas may cause accelerated biological aging.^10,13,28,29^ However, studies that directly link loss specifically with epigenetic-clock measures of aging are scarce. Most research has focused on early-life adversity or adverse childhood experiences based on review of existing research,^10,13,28^ with only 2 prior studies^50,51^ specifically examining loss of a parent or loved one. Of these, 1 considered only first-generation epigenetic clocks and found no association with loss, consistent with our analysis of the Horvath clock.^50^ While highly precise in predicting chronological age, the Horvath clock and other first-generation clocks tend to show lesser and inconsistent associations with morbidity and mortality and low sensitivity to stressors.^22,24,28,52,53^ The other available study concerned a small sample of older adults in Ireland.^51^ Our study therefore contributes evidence from a large, US population-based sample observed relatively early in the life course when experiences of loss may contribute to an acceleration of biological processes of aging.

We found that adult losses showed greater associations with biological aging markers than childhood or adolescence losses, a pattern also seen in a smaller sample of older adults in the Ireland study.^51^ This may be due to stronger adult reactions to traumatic events, impacting physiological risk, or greater epigenetic recovery from losses that occurred in the more distant past. Understanding the distinct impacts of loss at different life stages requires studies with repeated measures of loss and DNA methylation over time.

Several studies have highlighted significant disparities in loss among Black and Hispanic populations compared with White populations in the US.^3,6,14,40,41,42,43^ Consistent with recent work by Donnelly et al,^14^ we noted that Hispanic participants experienced a higher number of losses than White non-Hispanic participants. American Indian and Pacific Islanders also experienced greater loss, but the sample size for these estimates were small. Our interaction analysis suggests that there were no significant differences between loss and epigenetic aging across Black and White participants. A previous study noted higher instances and impacts of loss on GrimAge among Black participants.^54^ We were unable to stably test for effect modification across the smaller samples of racial and ethnic groups. Future research should examine these associations among American Indian, Hispanic, and Pacific Islanders, as well as individuals of multiracial and other identities.

### Limitations

We acknowledge limitations. We had access to only 1 time point of epigenetic data, leaving open questions as to why childhood or adolescent losses exhibited lesser associations with epigenetic clock measures as compared with losses occurring during adulthood, closer to the time of DNA collection. It is also possible that individuals with parents who die prematurely may inherit familial health conditions that impact both parental loss and biological aging. However, we adjusted for time since loss and did not observe significant differences in model estimates. This study may not have had sufficient power to detect minor interaction effects by race. Given the significant differences in exposure to loss across racial and ethnic groups, future research will require larger and more diverse population samples. The consistency of our results across 3 different epigenetic clocks and multiple model specifications in a large, diverse, prospective, national cohort contribute important evidence that experiences of loss represent a marker of risk for accelerated biological aging. Future research should aim to replicate our innovative findings related to the quantity of loss and its variability by type.

## Conclusions

In conclusion, our study sheds light on how loss may affect biological aging and ultimately health and mortality. We found that adults with a history of loss had higher biological ages than those without such experiences. More losses were associated with older biological age. These findings suggest that loss can accelerate biological aging even before midlife and frequency of losses may compound this, potentially leading to earlier chronic diseases and mortality. Future research should focus on identifying coping strategies and social support to lessen the negative effects of loss on aging, aiding clinical and public health approaches.

## Notes

Supplement 1. **eTable 1. **Number of Losses and Biological Aging **eTable 2. **Parental Loss at Any Time Period (Childhood to Adulthood) and Biological Aging **eTable 3. **Any Loss in Childhood and in Adulthood and Biological Aging **eTable 4. **Parental Loss in Childhood and in Adulthood and Biological Aging **eTable 5. **Count of Losses and Biological Aging **eTable 6. **Parental Loss at Any Time Period (Childhood to Adulthood) and Biological Aging **eTable 7. **Any Loss in Childhood and in Adulthood and Biological Aging **eTable 8. **Parental Loss in Childhood and in Adulthood and Biological Aging **eTable 9. **Number of Losses and Biological Aging **eTable 10. **Parental Loss at Any Time Period (Childhood to Adulthood) and Biological Aging **eTable 11. **Any Loss in Childhood and in Adulthood and Biological Aging **eTable 12. **Parental Loss in Childhood and in Adulthood and Biological Aging **eTable 13. **Number of Losses and Biological Aging **eTable 14. **Parental Loss at Any Time Period (Childhood to Adulthood) and Biological Aging **eTable 15. **Any Loss in Childhood and in Adulthood and Biological Aging **eTable 16. **Parental Loss in Childhood and in Adulthood and Biological Aging **eTable 17. **Parental Loss by Gender of Parent at Any Time Period (Childhood to Adulthood) and Biological Aging **eTable 18. **Interaction Between Any Loss in Childhood and in Adulthood and its Association With Biological **eTable 19. **Interaction Between Parental Loss in Childhood and in Adulthood and its Association With Biological Aging

Supplement 2.
Data Sharing Statement
