# Supplementary material for: Familial Loss of a Loved One and Biological Aging: NIMHD Social Epigenomics Program
Source: JAMA Netw Open. 2024 Jul 29;7(7):e2421869. doi: 10.1001/jamanetworkopen.2024.21869 (PMC11287397; doi:10.1001/jamanetworkopen.2024.21869)
Supplement: Supplement 1. — eTable 1. Number of Losses and Biological Aging eTable 2. Parental Loss at Any Time Period (Childhood to Adulthood) and Biological Aging eTable 3. Any Loss in Childhood and in Adulthood and Biological Aging eTable 4. Parental Loss in Childhood and in Adulthood and Biological Aging eTable 5. Count of Losses and Biological Aging eTable 6. Parental Loss at Any Time Period (Childhood to Adulthood) and Biological Aging eTable 7. Any Loss in Childhood and in Adulthood and Biological Aging eTable 8. Parental Loss in Childhood and in Adulthood and Biological Aging eTable 9. Number of Losses and Biological Aging eTable 10. Parental Loss at Any Time Period (Childhood to Adulthood) and Biological Aging eTable 11. Any Loss in Childhood and in Adulthood and Biological Aging eTable 12. Parental Loss in Childhood and in Adulthood and Biological Aging eTable 13. Number of Losses and Biological Aging eTable 14. Parental Loss at Any Time Period (Childhood to Adulthood) and Biological Aging eTable 15. Any Loss in Childhood and in Adulthood and Biological Aging eTable 16. Parental Loss in Childhood and in Adulthood and Biological Aging eTable 17. Parental Loss by Gender of Parent at Any Time Period (Childhood to Adulthood) and Biological Aging eTable 18. Interaction Between Any Loss in Childhood and in Adulthood and its Association With Biological eTable 19. Interaction Between Parental Loss in Childhood and in Adulthood and its Association With Biological Aging [file jamanetwopen-e2421869-s001.pdf]

Supplemental Online Content

Aiello AE, Mishra AA, Martin CL, et al. Familial loss of a loved one and biological aging: NIMHD social epigenomics program. *JAMA Netw Open*. 2024;7(7):e2421869.
doi:10.1001/jamanetworkopen.2024.21869

eTable 1. Number of Losses and Biological Aging

eTable 2. Parental Loss at Any Time Period (Childhood to Adulthood) and Biological Aging

eTable 3. Any Loss in Childhood and in Adulthood and Biological Aging

eTable 4. Parental Loss in Childhood and in Adulthood and Biological Aging

eTable 5. Count of Losses and Biological Aging

eTable 6. Parental Loss at Any Time Period (Childhood to Adulthood) and Biological Aging

eTable 7. Any Loss in Childhood and in Adulthood and Biological Aging

eTable 8. Parental Loss in Childhood and in Adulthood and Biological Aging

eTable 9. Number of Losses and Biological Aging

eTable 10. Parental Loss at Any Time Period (Childhood to Adulthood) and Biological Aging

eTable 11. Any Loss in Childhood and in Adulthood and Biological Aging

eTable 12. Parental Loss in Childhood and in Adulthood and Biological Aging

eTable 13. Number of Losses and Biological Aging

eTable 14. Parental Loss at Any Time Period (Childhood to Adulthood) and Biological Aging

eTable 15. Any Loss in Childhood and in Adulthood and Biological Aging

eTable 16. Parental Loss in Childhood and in Adulthood and Biological Aging

eTable 17. Parental Loss by Gender of Parent at Any Time Period (Childhood to Adulthood) and Biological Aging

eTable 18. Interaction Between Any Loss in Childhood and in Adulthood and its Association With Biological

eTable 19. Interaction Between Parental Loss in Childhood and in Adulthood and its Association With Biological Aging

© 2024 Aiello AE et al. *JAMA Network Open*.

This supplemental material has been provided by the authors to give readers additional information about their work.

© 2024 Aiello AE et al. *JAMA Network Open*.

eTable 1. Number of Losses and Biological Aging (*n* = 3,963)

Horvath						
	b	se	p		95% CI (Lower Limit)	95% CI (Upper Limit)
One Loss: Yes[a]	-0.01	0.05	0.784		-0.10	0.08
Two or More Losses: Yes[a]	-0.09	0.07	0.236		-0.23	0.06
Age	-0.02	0.01	0.185		-0.04	0.01
Race: Black[b]	0.01	0.08	0.920		-0.15	0.17
Race: Hispanic[b]	-0.19	0.10	0.069		-0.39	0.01
Race: Other[b,c]	0.02	0.09	0.802		-0.15	0.19
Parent Education: High School[d]	0.15	0.06	0.012	*	0.03	0.27
Parent Education: Some College[d]	0.07	0.05	0.179		-0.03	0.16
Gender: Female[e]	-0.34	0.05	0.000	***	-0.43	-0.25
Proportion of Households under Poverty	0.09	0.19	0.644		-0.29	0.46
No. of Household Members	0.01	0.02	0.718		-0.03	0.05
Caregiver Smokes: Yes[f]	-0.08	0.05	0.172		-0.18	0.03
Epigenetic Assay: Batch 1[g]	0.28	0.20	0.175		-0.13	0.68
Epigenetic Assay: Batch 2[g]	0.41	0.20	0.045	*	0.01	0.80
CD4T	-0.91	1.40	0.520		-3.69	1.88
CD8T	3.69	1.45	0.012	*	0.81	6.56
Bcell	0.37	1.36	0.788		-2.33	3.06
NK	3.13	1.64	0.058		-0.11	6.38
Mono	-0.97	1.74	0.580		-4.41	2.48
Gran	1.44	1.33	0.283		-1.20	4.08

PhenoAge						
	b	se	p		95% CI (Lower Limit)	95% CI (Upper Limit)
One Loss: Yes[a]	0.07	0.05	0.122		-0.02	0.16
Two or More Losses: Yes[a]	0.18	0.06	0.005	**	0.05	0.30
Age	-0.02	0.01	0.133		-0.04	0.01
Race: Black[b]	0.03	0.08	0.716		-0.12	0.18
Race: Hispanic[b]	0.04	0.09	0.631		-0.13	0.22
Race: Other[b,c]	0.06	0.10	0.582		-0.15	0.26
Parent Education: High School[d]	0.24	0.05	0.000	***	0.14	0.34
Parent Education: Some College[d]	0.15	0.05	0.006	**	0.05	0.26
Gender: Female[e]	0.10	0.04	0.016	*	0.02	0.18
Proportion of Households under Poverty	0.25	0.23	0.282		-0.20	0.70
No. of Household Members	-0.02	0.02	0.229		-0.06	0.01
Caregiver Smokes: Yes[f]	0.07	0.05	0.141		-0.02	0.17

© 2024 Aiello AE et al. *JAMA Network Open*.

	b	se	p		95% CI (Lower Limit)	95% CI (Upper Limit)
Epigenetic Assay: Batch 1[g]	-0.26	0.13	0.053		-0.53	0.00
Epigenetic Assay: Batch 2[g]	-0.23	0.14	0.088		-0.50	0.04
CD4T	1.01	1.39	0.466		-1.73	3.76
CD8T	1.11	1.34	0.410		-1.55	3.77
Bcell	1.06	1.62	0.516		-2.16	4.28
NK	6.01	1.58	0.000	***	2.88	9.15
Mono	6.90	1.77	0.000	***	3.39	10.40
Gran	5.86	1.34	0.000	***	3.20	8.52

GrimAge

	b	se	p		95% CI (Lower Limit)	95% CI (Upper Limit)
One Loss: Yes[a]	0.17	0.05	0.001	***	0.07	0.27
Two or More Losses: Yes[a]	0.27	0.08	0.001	**	0.11	0.44
Age	-0.05	0.01	0.000	***	-0.07	-0.03
Race: Black[b]	0.29	0.07	0.000	***	0.15	0.43
Race: Hispanic[b]	-0.34	0.08	0.000	***	-0.49	-0.18
Race: Other[b,c]	0.02	0.10	0.806		-0.16	0.21
Parent Education: High School[d]	0.37	0.06	0.000	***	0.26	0.48
Parent Education: Some College[d]	0.26	0.05	0.000	***	0.15	0.36
Gender: Female[e]	-0.49	0.04	0.000	***	-0.57	-0.41
Proportion of Households under Poverty	0.59	0.24	0.017	*	0.11	1.08
No. of Household Members	0.02	0.02	0.225		-0.02	0.06
Caregiver Smokes: Yes[f]	0.42	0.05	0.000	***	0.31	0.53
Epigenetic Assay: Batch 1[g]	-0.17	0.27	0.524		-0.71	0.37
Epigenetic Assay: Batch 2[g]	0.22	0.27	0.418		-0.31	0.75
CD4T	1.75	1.18	0.140		-0.58	4.08
CD8T	0.95	1.17	0.419		-1.37	3.26
Bcell	0.56	1.39	0.691		-2.21	3.32
NK	-0.86	1.35	0.526		-3.52	1.81
Mono	1.85	1.41	0.192		-0.94	4.65
Gran	3.66	1.12	0.001	**	1.44	5.89

DunedinPACE

	b	se	p		95% CI (Lower Limit)	95% CI (Upper Limit)
One Loss: Yes[a]	0.16	0.05	0.001	***	0.07	0.25
Two or More Losses: Yes[a]	0.22	0.06	0.000	***	0.11	0.34
Age	0.01	0.01	0.237		-0.01	0.04
Race: Black[b]	0.47	0.07	0.000	***	0.33	0.61
Race: Hispanic[b]	0.10	0.10	0.284		-0.09	0.30
Race: Other[b,c]	0.29	0.10	0.006	**	0.09	0.50

© 2024 Aiello AE et al. *JAMA Network Open*.

Parent Education: High School[d]	0.45	0.05	0.000	***	0.34	0.55
Parent Education: Some College[d]	0.26	0.05	0.000	***	0.15	0.36
Gender: Female[e]	0.11	0.04	0.007	**	0.03	0.19
Proportion of Households under Poverty	0.72	0.23	0.002	**	0.27	1.17
No. of Household Members	0.01	0.02	0.418		-0.02	0.05
Caregiver Smokes: Yes[f]	0.18	0.05	0.001	***	0.07	0.28
Epigenetic Assay: Batch 1[g]	0.02	0.12	0.887		-0.22	0.26
Epigenetic Assay: Batch 2[g]	0.08	0.12	0.487		-0.15	0.32
CD4T	-1.33	1.38	0.340		-4.06	1.41
CD8T	-1.91	1.30	0.146		-4.48	0.67
Bcell	3.70	1.53	0.017	*	0.67	6.73
NK	-2.02	1.49	0.177		-4.97	0.92
Mono	0.98	1.49	0.512		-1.97	3.93
Gran	2.35	1.33	0.079		-0.28	4.99

Note: a = No Loss (reference) b = White (reference); c = Asian, Native American, Pacific Islander, Other (other race or multi-racial); d = College or Higher reference; e = Male (reference); f = No (reference); g = Batch 3 (reference)

*$p \leq 0.05$; **$p \leq 0.01$; ***$p \leq 0.001$

NK - Natural Killer; Mono - Monocytes; Gran - Granulocytes

© 2024 Aiello AE et al. *JAMA Network Open*.

eTable 2. Parental Loss at Any Time Period (Childhood to Adulthood) and Biological Aging (*n* = 3,963)

Horvath						
	b	se	p		95% CI (Lower Limit)	95% CI (Upper Limit)
Parental Loss: Yes[a]	0.00	0.05	0.983		-0.10	0.09
Age	-0.02	0.01	0.147		-0.04	0.01
Race: Black[b]	0.00	0.08	0.997		-0.16	0.16
Race: Hispanic[b]	-0.18	0.10	0.076		-0.39	0.02
Race: Other[b,c]	0.02	0.08	0.801		-0.15	0.19
Parent Education: High School[d]	0.15	0.06	0.016	*	0.03	0.27
Parent Education: Some College[d]	0.06	0.05	0.202		-0.03	0.16
Gender: Female[e]	-0.34	0.05	0.000	***	-0.43	-0.25
Proportion of Households under Poverty	0.07	0.19	0.711		-0.30	0.44
No. of Household Members	0.01	0.02	0.700		-0.03	0.05
Caregiver Smokes: Yes[f]	-0.08	0.06	0.149		-0.19	0.03
Epigenetic Assay: Batch 1[g]	0.27	0.21	0.189		-0.14	0.68
Epigenetic Assay: Batch 2[g]	0.40	0.20	0.049	*	0.00	0.80
CD4T	-0.92	1.41	0.514		-3.71	1.87
CD8T	3.67	1.45	0.013	*	0.79	6.55
Bcell	0.33	1.37	0.808		-2.38	3.04
NK	3.12	1.64	0.060		-0.13	6.37
Mono	-0.96	1.74	0.583		-4.41	2.49
Gran	1.42	1.34	0.290		-1.23	4.07

PhenoAge						
	b	se	p		95% CI (Lower Limit)	95% CI (Upper Limit)
Parental Loss: Yes[a]	0.11	0.04	0.015	*	0.02	0.20
Age	-0.02	0.01	0.145		-0.04	0.01
Race: Black[b]	0.03	0.08	0.685		-0.12	0.18
Race: Hispanic[b]	0.04	0.09	0.685		-0.14	0.22
Race: Other[b,c]	0.05	0.10	0.616		-0.15	0.26
Parent Education: High School[d]	0.24	0.05	0.000	***	0.14	0.35
Parent Education: Some College[d]	0.16	0.05	0.005	**	0.05	0.26
Gender: Female[e]	0.10	0.04	0.016	*	0.02	0.18
Proportion of Households under Poverty	0.26	0.23	0.258		-0.19	0.71
No. of Household Members	-0.02	0.02	0.269		-0.06	0.02
Caregiver Smokes: Yes[f]	0.07	0.05	0.135		-0.02	0.17
Epigenetic Assay: Batch 1[g]	-0.27	0.13	0.050		-0.53	0.00
Epigenetic Assay: Batch 2[g]	-0.24	0.14	0.082		-0.51	0.03
CD4T	1.05	1.39	0.450		-1.70	3.81
CD8T	1.16	1.35	0.389		-1.50	3.83

© 2024 Aiello AE et al. *JAMA Network Open*.

	b	se	p		95% CI (Lower Limit)	95% CI (Upper Limit)
Bcell	1.16	1.63	0.478		-2.07	4.39
NK	6.07	1.58	0.000	***	2.93	9.21
Mono	6.90	1.78	0.000	***	3.38	10.42
Gran	5.91	1.35	0.000	***	3.24	8.58

GrimAge

	b	se	p		95% CI (Lower Limit)	95% CI (Upper Limit)
Parental Loss: Yes[a]	0.20	0.05	0.000	***	0.10	0.29
Age	-0.05	0.01	0.000	***	-0.07	-0.03
Race: Black[b]	0.30	0.07	0.000	***	0.16	0.43
Race: Hispanic[b]	-0.34	0.08	0.000	***	-0.50	-0.18
Race: Other[b,c]	0.02	0.10	0.835		-0.17	0.21
Parent Education: High School[d]	0.38	0.06	0.000	***	0.27	0.50
Parent Education: Some College[d]	0.26	0.05	0.000	***	0.16	0.37
Gender: Female[e]	-0.49	0.04	0.000	***	-0.57	-0.41
Proportion of Households under Poverty	0.61	0.25	0.015	*	0.12	1.10
No. of Household Members	0.03	0.02	0.182		-0.01	0.07
Caregiver Smokes: Yes[f]	0.42	0.05	0.000	***	0.31	0.53
Epigenetic Assay: Batch 1[g]	-0.18	0.27	0.500		-0.72	0.35
Epigenetic Assay: Batch 2[g]	0.21	0.26	0.435		-0.32	0.73
CD4T	1.79	1.17	0.130		-0.53	4.11
CD8T	1.02	1.16	0.381		-1.28	3.32
Bcell	0.69	1.41	0.624		-2.09	3.48
NK	-0.80	1.33	0.550		-3.44	1.84
Mono	1.84	1.42	0.197		-0.97	4.64
Gran	3.73	1.12	0.001	**	1.52	5.94

DunedinPACE

	b	se	p		95% CI (Lower Limit)	95% CI (Upper Limit)
Parental Loss: Yes[a]	0.19	0.04	0.000	***	0.10	0.27
Age	0.01	0.01	0.223		-0.01	0.04
Race: Black[b]	0.47	0.07	0.000	***	0.33	0.62
Race: Hispanic[b]	0.10	0.10	0.307		-0.10	0.30
Race: Other[b,c]	0.29	0.10	0.006	**	0.08	0.49
Parent Education: High School[d]	0.46	0.05	0.000	***	0.35	0.56
Parent Education: Some College[d]	0.26	0.05	0.000	***	0.16	0.36
Gender: Female[e]	0.11	0.04	0.007	**	0.03	0.19
Proportion of Households under Poverty	0.73	0.23	0.002	**	0.28	1.18
No. of Household Members	0.02	0.02	0.346		-0.02	0.05
Caregiver Smokes: Yes[f]	0.18	0.05	0.001	***	0.07	0.28
Epigenetic Assay: Batch 1[g]	0.01	0.12	0.958		-0.23	0.25

© 2024 Aiello AE et al. *JAMA Network Open*.

Epigenetic Assay: Batch 2[g]	0.07	0.12	0.545		-0.16	0.31
CD4T	-1.29	1.38	0.350		-4.03	1.44
CD8T	-1.84	1.30	0.159		-4.42	0.73
Bcell	3.81	1.54	0.015	*	0.77	6.86
NK	-1.98	1.48	0.185		-4.92	0.96
Mono	0.97	1.49	0.519		-1.99	3.93
Gran	2.40	1.33	0.073		-0.22	5.03

Note: a = No Loss (reference) b = White (reference); c = Asian, Native American, Pacific Islander, Other (other race or multi-racial); d = College or Higher reference; e = Male (reference); f = No (reference); g = Batch 3 (reference)

*$p \leq 0.05$; **$p \leq 0.01$; ***$p \leq 0.001$

NK - Natural Killer; Mono - Monocytes; Gran - Granulocytes

© 2024 Aiello AE et al. *JAMA Network Open*.

eTable 3. Any Loss in Childhood and in Adulthood and Biological Aging (*n* = 3,963)

	b	se	p		95% CI (Lower Limit)	95% CI (Upper Limit)
**Horvath**						
Any Loss in Childhood: Yes[a]	-0.04	0.07	0.561		-0.19	0.10
Any Loss in Adulthood: Yes[a]	-0.01	0.05	0.824		-0.11	0.09
Age	-0.02	0.01	0.152		-0.04	0.01
Race: Black[b]	0.00	0.08	0.964		-0.16	0.16
Race: Hispanic[b]	-0.18	0.10	0.075		-0.39	0.02
Race: Other[b,c]	0.02	0.09	0.792		-0.15	0.19
Parent Education: High School[d]	0.15	0.06	0.015	*	0.03	0.27
Parent Education: Some College[d]	0.06	0.05	0.203		-0.03	0.16
Gender: Female[e]	-0.34	0.05	0.000	***	-0.43	-0.25
Proportion of Households under Poverty	0.08	0.19	0.683		-0.29	0.45
No. of Household Members	0.01	0.02	0.720		-0.03	0.05
Caregiver Smokes: Yes[f]	-0.08	0.06	0.161		-0.19	0.03
Epigenetic Assay: Batch 1[g]	0.27	0.21	0.186		-0.13	0.68
Epigenetic Assay: Batch 2[g]	0.40	0.20	0.048	*	0.00	0.80
CD4T	-0.91	1.40	0.516		-3.69	1.87
CD8T	3.67	1.45	0.013	*	0.79	6.54
Bcell	0.35	1.37	0.799		-2.36	3.06
NK	3.12	1.64	0.059		-0.12	6.36
Mono	-0.96	1.74	0.582		-4.41	2.49
Gran	1.42	1.33	0.288		-1.22	4.06
**PhenoAge**						
	b	se	p		95% CI (Lower Limit)	95% CI (Upper Limit)
Any Loss in Childhood: Yes[a]	0.12	0.07	0.084		-0.02	0.26
Any Loss in Adulthood: Yes[a]	0.08	0.05	0.082		-0.01	0.18
Age	-0.02	0.01	0.163		-0.04	0.01
Race: Black[b]	0.03	0.08	0.675		-0.12	0.18
Race: Hispanic[b]	0.04	0.09	0.660		-0.14	0.22
Race: Other[b,c]	0.06	0.11	0.591		-0.15	0.27
Parent Education: High School[d]	0.24	0.05	0.000	***	0.14	0.34
Parent Education: Some College[d]	0.16	0.05	0.004	**	0.05	0.26
Gender: Female[e]	0.10	0.04	0.019	*	0.02	0.18
Proportion of Households under Poverty	0.25	0.23	0.266		-0.19	0.70
No. of Household Members	-0.02	0.02	0.254		-0.06	0.02
Caregiver Smokes: Yes[f]	0.07	0.05	0.140		-0.02	0.17

© 2024 Aiello AE et al. *JAMA Network Open*.

	b	se	p		95% CI (Lower Limit)	95% CI (Upper Limit)
Epigenetic Assay: Batch 1[g]	-0.26	0.13	0.057		-0.52	0.01
Epigenetic Assay: Batch 2[g]	-0.23	0.14	0.093		-0.50	0.04
CD4T	1.03	1.38	0.458		-1.71	3.76
CD8T	1.16	1.34	0.391		-1.50	3.82
Bcell	1.10	1.62	0.499		-2.11	4.30
NK	6.05	1.58	0.000	***	2.93	9.17
Mono	6.90	1.77	0.000	***	3.39	10.42
Gran	5.89	1.34	0.000	***	3.24	8.55

GrimAge

	b	se	p		95% CI (Lower Limit)	95% CI (Upper Limit)
Any Loss in Childhood: Yes[a]	0.13	0.09	0.126		-0.04	0.30
Any Loss in Adulthood: Yes[a]	0.15	0.05	0.003	**	0.05	0.25
Age	-0.05	0.01	0.000	***	-0.07	-0.03
Race: Black[b]	0.30	0.07	0.000	***	0.17	0.44
Race: Hispanic[b]	-0.33	0.08	0.000	***	-0.49	-0.18
Race: Other[b,c]	0.03	0.10	0.758		-0.16	0.22
Parent Education: High School[d]	0.38	0.06	0.000	***	0.27	0.50
Parent Education: Some College[d]	0.27	0.05	0.000	***	0.16	0.37
Gender: Female[e]	-0.50	0.04	0.000	***	-0.58	-0.42
Proportion of Households under Poverty	0.60	0.24	0.015	*	0.12	1.09
No. of Household Members	0.03	0.02	0.214		-0.01	0.06
Caregiver Smokes: Yes[f]	0.42	0.06	0.000	***	0.31	0.53
Epigenetic Assay: Batch 1[g]	-0.17	0.28	0.548		-0.71	0.38
Epigenetic Assay: Batch 2[g]	0.22	0.27	0.403		-0.31	0.76
CD4T	1.77	1.19	0.141		-0.60	4.13
CD8T	1.01	1.18	0.394		-1.33	3.36
Bcell	0.63	1.43	0.660		-2.20	3.46
NK	-0.82	1.36	0.549		-3.51	1.87
Mono	1.85	1.42	0.195		-0.96	4.66
Gran	3.72	1.14	0.001	**	1.46	5.97

DunedinPACE

	b	se	p		95% CI (Lower Limit)	95% CI (Upper Limit)
Any Loss in Childhood: Yes[a]	0.09	0.08	0.271		-0.07	0.26
Any Loss in Adulthood: Yes[a]	0.14	0.04	0.002	**	0.06	0.23
Age	0.01	0.01	0.207		-0.01	0.04
Race: Black[b]	0.48	0.07	0.000	***	0.34	0.62
Race: Hispanic[b]	0.11	0.10	0.280		-0.09	0.30
Race: Other[b,c]	0.30	0.11	0.006	**	0.09	0.51

Parent Education: High School[d]	0.46	0.05	0.000	***	0.35	0.56
Parent Education: Some College[d]	0.26	0.05	0.000	***	0.16	0.36
Gender: Female[e]	0.11	0.04	0.007	**	0.03	0.19
Proportion of Households under Poverty	0.73	0.23	0.002	**	0.28	1.17
No. of Household Members	0.01	0.02	0.406		-0.02	0.05
Caregiver Smokes: Yes[f]	0.18	0.05	0.001	***	0.08	0.28
Epigenetic Assay: Batch 1[g]	0.02	0.12	0.855		-0.22	0.27
Epigenetic Assay: Batch 2[g]	0.09	0.12	0.465		-0.15	0.32
CD4T	-1.30	1.39	0.352		-4.06	1.46
CD8T	-1.85	1.32	0.162		-4.46	0.76
Bcell	3.78	1.55	0.016	*	0.71	6.84
NK	-1.98	1.50	0.190		-4.96	0.99
Mono	0.98	1.50	0.514		-1.99	3.96
Gran	2.40	1.34	0.075		-0.25	5.06

Note: a = No Loss (reference) b = White (reference); c = Asian, Native American, Pacific Islander, Other (other race or multi-racial); d = College or Higher reference; e = Male (reference); f = No (reference); g = Batch 3 (reference)

*$p \leq 0.05$; **$p \leq 0.01$; ***$p \leq 0.001$

NK - Natural Killer; Mono - Monocytes; Gran - Granulocytes

© 2024 Aiello AE et al. *JAMA Network Open*.

eTable 4. Parental Loss in Childhood and in Adulthood and Biological Aging (*n* = 3,963)

Horvath						
	b	se	p		95% CI (Lower Limit)	95% CI (Upper Limit)
Parental Loss in Childhood: Yes[a]	-0.05	0.08	0.508		-0.21	0.11
Parental Loss in Adulthood: Yes[a]	0.02	0.05	0.658		-0.08	0.12
Age	-0.02	0.01	0.133		-0.04	0.01
Race: Black[b]	0.00	0.08	0.999		-0.16	0.16
Race: Hispanic[b]	-0.18	0.10	0.077		-0.39	0.02
Race: Other[b,c]	0.02	0.08	0.794		-0.15	0.19
Parent Education: High School[d]	0.15	0.06	0.016	*	0.03	0.27
Parent Education: Some College[d]	0.06	0.05	0.214		-0.04	0.16
Gender: Female[e]	-0.34	0.05	0.000	***	-0.43	-0.25
Proportion of Households under Poverty	0.07	0.19	0.724		-0.31	0.44
No. of Household Members	0.01	0.02	0.701		-0.03	0.05
Caregiver Smokes: Yes[f]	-0.08	0.06	0.143		-0.19	0.03
Epigenetic Assay: Batch 1[g]	0.27	0.20	0.193		-0.14	0.67
Epigenetic Assay: Batch 2[g]	0.40	0.20	0.051		0.00	0.80
CD4T	-0.89	1.40	0.526		-3.68	1.89
CD8T	3.67	1.45	0.013	*	0.80	6.55
Bcell	0.37	1.37	0.787		-2.34	3.08
NK	3.16	1.64	0.056		-0.09	6.40
Mono	-0.94	1.74	0.590		-4.39	2.51
Gran	1.44	1.33	0.283		-1.20	4.08

PhenoAge						
	b	se	p		95% CI (Lower Limit)	95% CI (Upper Limit)
Parental Loss in Childhood: Yes[a]	0.10	0.08	0.182		-0.05	0.25
Parental Loss in Adulthood: Yes[a]	0.10	0.05	0.043	*	0.00	0.19
Age	-0.02	0.01	0.157		-0.04	0.01
Race: Black[b]	0.03	0.08	0.662		-0.12	0.18
Race: Hispanic[b]	0.04	0.09	0.678		-0.14	0.22
Race: Other[b,c]	0.06	0.11	0.599		-0.15	0.26
Parent Education: High School[d]	0.24	0.05	0.000	***	0.14	0.34
Parent Education: Some College[d]	0.16	0.05	0.004	**	0.05	0.26
Gender: Female[e]	0.10	0.04	0.017	*	0.02	0.18
Proportion of Households under Poverty	0.26	0.23	0.252		-0.19	0.71
No. of Household Members	-0.02	0.02	0.271		-0.06	0.02
Caregiver Smokes: Yes[f]	0.07	0.05	0.136		-0.02	0.17
Epigenetic Assay: Batch 1[g]	-0.26	0.13	0.052		-0.53	0.00

© 2024 Aiello AE et al. *JAMA Network Open*.

Epigenetic Assay: Batch 2[g]	-0.24	0.14	0.085		-0.51	0.03
CD4T	1.04	1.38	0.452		-1.69	3.78
CD8T	1.17	1.34	0.385		-1.49	3.83
Bcell	1.15	1.62	0.481		-2.07	4.36
NK	6.06	1.58	0.000	***	2.93	9.18
Mono	6.91	1.77	0.000	***	3.40	10.41
Gran	5.91	1.34	0.000	***	3.25	8.56

GrimAge

	b	se	p		95% CI (Lower Limit)	95% CI (Upper Limit)
Parental Loss in Childhood: Yes[a]	0.12	0.10	0.196		-0.07	0.31
Parental Loss in Adulthood: Yes[a]	0.15	0.05	0.003	**	0.05	0.25
Age	-0.05	0.01	0.000	***	-0.07	-0.03
Race: Black[b]	0.31	0.07	0.000	***	0.17	0.44
Race: Hispanic[b]	-0.34	0.08	0.000	***	-0.49	-0.18
Race: Other[b,c]	0.03	0.10	0.775		-0.16	0.22
Parent Education: High School[d]	0.38	0.06	0.000	***	0.27	0.50
Parent Education: Some College[d]	0.27	0.05	0.000	***	0.16	0.37
Gender: Female[e]	-0.50	0.04	0.000	***	-0.58	-0.42
Proportion of Households under Poverty	0.62	0.25	0.014	*	0.13	1.11
No. of Household Members	0.03	0.02	0.204		-0.01	0.07
Caregiver Smokes: Yes[f]	0.42	0.06	0.000	***	0.31	0.53
Epigenetic Assay: Batch 1[g]	-0.17	0.27	0.530		-0.71	0.37
Epigenetic Assay: Batch 2[g]	0.22	0.27	0.414		-0.31	0.74
CD4T	1.77	1.19	0.138		-0.58	4.12
CD8T	1.03	1.17	0.382		-1.29	3.35
Bcell	0.66	1.43	0.643		-2.16	3.49
NK	-0.83	1.35	0.539		-3.51	1.84
Mono	1.84	1.42	0.196		-0.96	4.64
Gran	3.72	1.13	0.001	**	1.48	5.96

DunedinPACE

	b	se	p		95% CI (Lower Limit)	95% CI (Upper Limit)
Parental Loss in Childhood: Yes[a]	0.10	0.09	0.280		-0.08	0.27
Parental Loss in Adulthood: Yes[a]	0.16	0.04	0.000	***	0.07	0.25
Age	0.01	0.01	0.210		-0.01	0.04
Race: Black[b]	0.48	0.07	0.000	***	0.34	0.62
Race: Hispanic[b]	0.11	0.10	0.295		-0.09	0.30
Race: Other[b,c]	0.29	0.10	0.006	**	0.09	0.50
Parent Education: High School[d]	0.46	0.05	0.000	***	0.35	0.56
Parent Education: Some College[d]	0.26	0.05	0.000	***	0.16	0.36

© 2024 Aiello AE et al. *JAMA Network Open*.

Gender: Female[e]	0.11	0.04	0.007	**	0.03	0.19
Proportion of Households under Poverty	0.74	0.23	0.002	**	0.29	1.18
No. of Household Members	0.02	0.02	0.380		-0.02	0.05
Caregiver Smokes: Yes[f]	0.18	0.05	0.001	***	0.08	0.28
Epigenetic Assay: Batch 1[g]	0.01	0.12	0.909		-0.23	0.25
Epigenetic Assay: Batch 2[g]	0.08	0.12	0.506		-0.15	0.31
CD4T	-1.30	1.39	0.353		-4.05	1.46
CD8T	-1.83	1.31	0.164		-4.42	0.76
Bcell	3.81	1.54	0.015	*	0.75	6.87
NK	-1.99	1.50	0.188		-4.96	0.98
Mono	0.98	1.50	0.515		-1.99	3.95
Gran	2.41	1.34	0.074		-0.23	5.06

Note: a = No Loss (reference) b = White (reference); Asian, Native American, Pacific Islander, Other (other race or multi-racial); d = College or Higher reference; e = Male (reference); f = No (reference); g = Batch 3 (reference)

*$p \leq 0.05$; **$p \leq 0.01$; ***$p \leq 0.001$

NK - Natural Killer; Mono - Monocytes; Gran - Granulocytes

© 2024 Aiello AE et al. *JAMA Network Open*.

eTable 5. Count of Losses and Biological Aging (*n* = 3,921)						
Horvath						
	b	se	p		95% CI (Lower Limit)	95% CI (Upper Limit)
One Loss: Yes[a]	-0.03	0.05	0.562		-0.14	0.08
Two or More Losses: Yes[a]	-0.12	0.09	0.215		-0.30	0.07
Age	-0.02	0.01	0.212		-0.04	0.01
Race: Black[b]	-0.02	0.08	0.835		-0.17	0.14
Race: Hispanic[b]	-0.16	0.10	0.106		-0.36	0.03
Race: Other[b,c]	0.05	0.10	0.607		-0.15	0.26
Parent Education: High School[d]	0.13	0.06	0.045	*	0.00	0.25
Parent Education: Some College[d]	0.02	0.05	0.692		-0.08	0.11
Gender: Female[e]	-0.37	0.04	0.000	***	-0.45	-0.29
Proportion of Households under Poverty	0.12	0.20	0.543		-0.27	0.52
No. of Household Members	0.01	0.02	0.468		-0.03	0.06
Caregiver Smokes: Yes[f]	-0.07	0.06	0.241		-0.19	0.05
Epigenetic Assay: Batch 1[g]	0.33	0.24	0.160		-0.13	0.80
Epigenetic Assay: Batch 2[g]	0.40	0.23	0.084		-0.06	0.86
Time Since Loss	0.00	0.00	0.480		-0.01	0.01
PhenoAge						
	b	se	p		95% CI (Lower Limit)	95% CI (Upper Limit)
One Loss: Yes[a]	0.04	0.06	0.529		-0.09	0.17
Two or More Losses: Yes[a]	0.09	0.08	0.274		-0.07	0.25
Age	-0.01	0.01	0.505		-0.03	0.02
Race: Black[b]	-0.20	0.07	0.006	**	-0.34	-0.06
Race: Hispanic[b]	-0.04	0.10	0.684		-0.24	0.16
Race: Other[b,c]	-0.05	0.13	0.689		-0.32	0.21
Parent Education: High School[d]	0.27	0.05	0.000	***	0.16	0.37
Parent Education: Some College[d]	0.17	0.06	0.006	**	0.05	0.29
Gender: Female[e]	0.10	0.04	0.010	*	0.03	0.18
Proportion of Households under Poverty	0.17	0.24	0.462		-0.29	0.64
No. of Household Members	-0.01	0.02	0.612		-0.05	0.03
Caregiver Smokes: Yes[f]	0.08	0.05	0.138		-0.02	0.18
Epigenetic Assay: Batch 1[g]	-0.15	0.15	0.338		-0.45	0.15
Epigenetic Assay: Batch 2[g]	-0.16	0.16	0.322		-0.46	0.15
Time Since Loss	0.01	0.00	0.176		0.00	0.01
GrimAge						

© 2024 Aiello AE et al. *JAMA Network Open*.

	b	se	p		95% CI (Lower Limit)	95% CI (Upper Limit)
One Loss: Yes[a]	0.17	0.07	0.011	*	0.04	0.31
Two or More Losses: Yes[a]	0.24	0.09	0.011	*	0.06	0.43
Age	-0.05	0.01	0.000	***	-0.08	-0.03
Race: Black[b]	0.12	0.06	0.075		-0.01	0.24
Race: Hispanic[b]	-0.42	0.09	0.000	***	-0.59	-0.24
Race: Other[b,c]	-0.06	0.10	0.503		-0.25	0.12
Parent Education: High School[d]	0.41	0.06	0.000	***	0.29	0.53
Parent Education: Some College[d]	0.29	0.05	0.000	***	0.18	0.40
Gender: Female[e]	-0.39	0.04	0.000	***	-0.47	-0.30
Proportion of Households under Poverty	0.46	0.25	0.063		-0.02	0.95
No. of Household Members	0.02	0.02	0.294		-0.02	0.07
Caregiver Smokes: Yes[f]	0.44	0.06	0.000	***	0.33	0.55
Epigenetic Assay: Batch 1[g]	-0.17	0.28	0.556		-0.72	0.39
Epigenetic Assay: Batch 2[g]	0.21	0.28	0.456		-0.34	0.75
Time Since Loss	0.00	0.00	0.873		-0.01	0.01

DunedinPACE

	b	se	p		95% CI (Lower Limit)	95% CI (Upper Limit)
One Loss: Yes[a]	0.17	0.06	0.010	*	0.04	0.30
Two or More Losses: Yes[a]	0.21	0.07	0.006	**	0.06	0.35
Age	0.02	0.01	0.144		-0.01	0.04
Race: Black[b]	0.35	0.06	0.000	***	0.22	0.48
Race: Hispanic[b]	0.06	0.11	0.546		-0.15	0.27
Race: Other[b,c]	0.25	0.12	0.033	*	0.02	0.48
Parent Education: High School[d]	0.51	0.05	0.000	***	0.40	0.62
Parent Education: Some College[d]	0.29	0.05	0.000	***	0.19	0.40
Gender: Female[e]	0.17	0.04	0.000	***	0.09	0.26
Proportion of Households under Poverty	0.63	0.23	0.007	**	0.18	1.09
No. of Household Members	0.02	0.02	0.404		-0.02	0.05
Caregiver Smokes: Yes[f]	0.19	0.06	0.002	**	0.07	0.30
Epigenetic Assay: Batch 1[g]	0.06	0.12	0.613		-0.18	0.31
Epigenetic Assay: Batch 2[g]	0.14	0.12	0.244		-0.10	0.38
Time Since Loss	0.00	0.00	0.956		-0.01	0.01

Note: a = No Loss (reference) b = White (reference); c = Asian, Native American, Pacific Islander, Other (other race or multi-racial); d = College or Higher reference; e = Male (reference); f = No (reference); g = Batch 3 (reference)

*$p \leq 0.05$; **$p \leq 0.01$; ***$p \leq 0.001$

© 2024 Aiello AE et al. *JAMA Network Open*.

eTable 6. Parental Loss at Any Time Period (Childhood to Adulthood) and Biological Aging (*n* = 3,921)

**Horvath**						
	b	se	p		95% CI (Lower Limit)	95% CI (Upper Limit)
Parental Loss: Yes[a]	-0.01	0.06	0.930		-0.13	0.12
Age	-0.02	0.01	0.177		-0.04	0.01
Race: Black[b]	-0.02	0.08	0.773		-0.18	0.13
Race: Hispanic[b]	-0.16	0.10	0.116		-0.35	0.04
Race: Other[b,c]	0.05	0.10	0.605		-0.15	0.26
Parent Education: High School[d]	0.12	0.06	0.055		0.00	0.24
Parent Education: Some College[d]	0.02	0.05	0.747		-0.08	0.11
Gender: Female[e]	-0.37	0.04	0.000	***	-0.45	-0.29
Proportion of Households under Poverty	0.11	0.20	0.592		-0.29	0.50
No. of Household Members	0.01	0.02	0.476		-0.03	0.06
Caregiver Smokes: Yes[f]	-0.07	0.06	0.214		-0.19	0.04
Epigenetic Assay: Batch 1[g]	0.33	0.24	0.167		-0.14	0.79
Epigenetic Assay: Batch 2[g]	0.40	0.23	0.089		-0.06	0.85
Time Since Loss	0.00	0.00	0.919		-0.01	0.01
**PhenoAge**						
	b	se	p		95% CI (Lower Limit)	95% CI (Upper Limit)
Parental Loss: Yes[a]	0.06	0.07	0.349		-0.07	0.20
Age	-0.01	0.01	0.519		-0.03	0.02
Race: Black[b]	-0.20	0.07	0.007	**	-0.34	-0.06
Race: Hispanic[b]	-0.04	0.10	0.674		-0.25	0.16
Race: Other[b,c]	-0.05	0.14	0.687		-0.32	0.21
Parent Education: High School[d]	0.27	0.05	0.000	***	0.16	0.38
Parent Education: Some College[d]	0.17	0.06	0.005	**	0.05	0.29
Gender: Female[e]	0.10	0.04	0.011	*	0.02	0.19
Proportion of Households under Poverty	0.18	0.24	0.451		-0.29	0.65
No. of Household Members	-0.01	0.02	0.644		-0.05	0.03
Caregiver Smokes: Yes[f]	0.08	0.05	0.140		-0.03	0.18
Epigenetic Assay: Batch 1[g]	-0.15	0.15	0.332		-0.45	0.15
Epigenetic Assay: Batch 2[g]	-0.16	0.16	0.312		-0.47	0.15
Time Since Loss	0.00	0.00	0.264		0.00	0.01
**GrimAge**						

© 2024 Aiello AE et al. *JAMA Network Open*.

	b	se	p		95% CI (Lower Limit)	95% CI (Upper Limit)
Parental Loss: Yes[a]	0.20	0.07	0.006	**	0.06	0.34
Age	-0.05	0.01	0.000	***	-0.08	-0.03
Race: Black[b]	0.12	0.06	0.057		0.00	0.25
Race: Hispanic[b]	-0.41	0.09	0.000	***	-0.60	-0.23
Race: Other[b,c]	-0.06	0.10	0.513		-0.25	0.13
Parent Education: High School[d]	0.42	0.06	0.000	***	0.30	0.55
Parent Education: Some College[d]	0.29	0.05	0.000	***	0.19	0.40
Gender: Female[e]	-0.39	0.04	0.000	***	-0.47	-0.30
Proportion of Households under Poverty	0.47	0.25	0.060		-0.02	0.96
No. of Household Members	0.03	0.02	0.251		-0.02	0.07
Caregiver Smokes: Yes[f]	0.44	0.06	0.000	***	0.33	0.55
Epigenetic Assay: Batch 1[g]	-0.17	0.28	0.533		-0.72	0.38
Epigenetic Assay: Batch 2[g]	0.20	0.27	0.472		-0.34	0.74
Time Since Loss	0.00	0.00	0.960		-0.01	0.01

DunedinPACE

	b	se	p		95% CI (Lower Limit)	95% CI (Upper Limit)
Parental Loss: Yes[a]	0.21	0.07	0.003	**	0.07	0.35
Age	0.02	0.01	0.134		-0.01	0.04
Race: Black[b]	0.35	0.06	0.000	***	0.22	0.48
Race: Hispanic[b]	0.07	0.11	0.553		-0.15	0.29
Race: Other[b,c]	0.25	0.12	0.032	*	0.02	0.49
Parent Education: High School[d]	0.52	0.06	0.000	***	0.41	0.63
Parent Education: Some College[d]	0.30	0.05	0.000	***	0.19	0.40
Gender: Female[e]	0.17	0.04	0.000	***	0.09	0.26
Proportion of Households under Poverty	0.64	0.23	0.007	**	0.18	1.09
No. of Household Members	0.02	0.02	0.341		-0.02	0.06
Caregiver Smokes: Yes[f]	0.19	0.06	0.002	**	0.07	0.30
Epigenetic Assay: Batch 1[g]	0.05	0.12	0.673		-0.19	0.30
Epigenetic Assay: Batch 2[g]	0.13	0.12	0.287		-0.11	0.36
Time Since Loss	0.00	0.00	0.681		-0.01	0.01

Note: a = No Loss (reference) b = White (reference); c = Asian, Native American, Pacific Islander, Other (other race or multi-racial); d = College or Higher reference; e = Male (reference); f = No (reference); g = Batch 3 (reference)

*$p \leq 0.05$; **$p \leq 0.01$; ***$p \leq 0.001$

© 2024 Aiello AE et al. *JAMA Network Open*.

eTable 7. Any Loss in Childhood and in Adulthood and Biological Aging (*n* = 3,921)						
Horvath						
	b	se	p		95% CI (Lower Limit)	95% CI (Upper Limit
Any Loss in Childhood: Yes[a]	-0.17	0.12	0.160		-0.40	0.07
Any Loss in Adulthood: Yes[a]	-0.04	0.05	0.445		-0.15	0.07
Age	-0.02	0.01	0.161		-0.04	0.01
Race: Black[b]	-0.02	0.08	0.791		-0.18	0.13
Race: Hispanic[b]	-0.16	0.10	0.114		-0.35	0.04
Race: Other[b,c]	0.06	0.10	0.591		-0.15	0.26
Parent Education: High School[d]	0.12	0.06	0.050	*	0.00	0.25
Parent Education: Some College[d]	0.01	0.05	0.758		-0.08	0.11
Gender: Female[e]	-0.37	0.04	0.000	***	-0.45	-0.29
Proportion of Households under Poverty	0.12	0.20	0.542		-0.27	0.51
No. of Household Members	0.01	0.02	0.478		-0.03	0.06
Caregiver Smokes: Yes[f]	-0.07	0.06	0.246		-0.19	0.05
Epigenetic Assay: Batch 1[g]	0.33	0.24	0.170		-0.14	0.79
Epigenetic Assay: Batch 2[g]	0.40	0.23	0.091		-0.06	0.86
Time Since Loss	0.01	0.00	0.242		0.00	0.02
PhenoAge						
	b	se	p		95% CI (Lower Limit)	95% CI (Upper Limit
Any Loss in Childhood: Yes[a]	-0.09	0.12	0.428		-0.32	0.14
Any Loss in Adulthood: Yes[a]	0.03	0.06	0.604		-0.08	0.14
Age	-0.01	0.01	0.484		-0.03	0.02
Race: Black[b]	-0.20	0.07	0.007	**	-0.34	-0.06
Race: Hispanic[b]	-0.04	0.10	0.679		-0.25	0.16
Race: Other[b,c]	-0.05	0.14	0.697		-0.32	0.22
Parent Education: High School[d]	0.27	0.05	0.000	***	0.16	0.38
Parent Education: Some College[d]	0.17	0.06	0.006	**	0.05	0.29
Gender: Female[e]	0.11	0.04	0.010	*	0.03	0.18
Proportion of Households under Poverty	0.18	0.24	0.440		-0.28	0.65
No. of Household Members	-0.01	0.02	0.638		-0.05	0.03
Caregiver Smokes: Yes[f]	0.08	0.05	0.124		-0.02	0.18
Epigenetic Assay: Batch 1[g]	-0.15	0.15	0.336		-0.45	0.15
Epigenetic Assay: Batch 2[g]	-0.16	0.16	0.319		-0.47	0.15
Time Since Loss	0.01	0.00	0.043	*	0.00	0.02
GrimAge						

© 2024 Aiello AE et al. *JAMA Network Open*.

	b	se	p		95% CI (Lower Limit)	95% CI (Upper Limit
Any Loss in Childhood: Yes[a]	0.06	0.11	0.554		-0.15	0.27
Any Loss in Adulthood: Yes[a]	0.16	0.06	0.008	**	0.04	0.28
Age	-0.05	0.01	0.000	***	-0.08	-0.03
Race: Black[b]	0.12	0.06	0.056		0.00	0.25
Race: Hispanic[b]	-0.41	0.09	0.000	***	-0.59	-0.23
Race: Other[b,c]	-0.06	0.10	0.521		-0.25	0.13
Parent Education: High School[d]	0.42	0.06	0.000	***	0.30	0.54
Parent Education: Some College[d]	0.29	0.05	0.000	***	0.19	0.40
Gender: Female[e]	-0.39	0.04	0.000	***	-0.47	-0.30
Proportion of Households under Poverty	0.46	0.25	0.065		-0.03	0.95
No. of Household Members	0.02	0.02	0.259		-0.02	0.07
Caregiver Smokes: Yes[f]	0.44	0.06	0.000	***	0.33	0.55
Epigenetic Assay: Batch 1[g]	-0.17	0.28	0.552		-0.72	0.39
Epigenetic Assay: Batch 2[g]	0.21	0.28	0.458		-0.34	0.75
Time Since Loss	0.00	0.00	0.439		-0.01	0.01

DunedinPACE

	b	se	p		95% CI (Lower Limit)	95% CI (Upper Limit
Any Loss in Childhood: Yes[a]	0.00	0.10	0.995		-0.20	0.20
Any Loss in Adulthood: Yes[a]	0.13	0.06	0.027	*	0.01	0.24
Age	0.02	0.01	0.147		-0.01	0.04
Race: Black[b]	0.35	0.06	0.000	***	0.23	0.48
Race: Hispanic[b]	0.07	0.11	0.529		-0.15	0.29
Race: Other[b,c]	0.26	0.12	0.033	*	0.02	0.49
Parent Education: High School[d]	0.52	0.06	0.000	***	0.41	0.63
Parent Education: Some College[d]	0.30	0.05	0.000	***	0.19	0.40
Gender: Female[e]	0.17	0.04	0.000	***	0.09	0.26
Proportion of Households under Poverty	0.63	0.23	0.007	**	0.18	1.09
No. of Household Members	0.02	0.02	0.363		-0.02	0.05
Caregiver Smokes: Yes[f]	0.19	0.06	0.001	**	0.07	0.31
Epigenetic Assay: Batch 1[g]	0.06	0.12	0.622		-0.18	0.31
Epigenetic Assay: Batch 2[g]	0.14	0.12	0.248		-0.10	0.37
Time Since Loss	0.00	0.00	0.235		0.00	0.01

Note: a = No Loss (reference) b = White (reference); c = Asian, Native American, Pacific Islander, Other (other race or multi-racial); d = College or Higher reference; e = Male (reference); f = No (reference); g = Batch 3 (reference)

*$p \leq 0.05$; **$p \leq 0.01$; ***$p \leq 0.001$

© 2024 Aiello AE et al. *JAMA Network Open*.

eTable 8. Parental Loss in Childhood and in Adulthood and Biological Aging (n = 3,921)						
**Horvath**						
	b	se	p		95% CI (Lower Limit)	95% CI (Upper Limit)
Parental Loss in Childhood: Yes[a]	-0.21	0.17	0.224		-0.54	0.13
Parental Loss in Adulthood: Yes[a]	-0.02	0.06	0.762		-0.14	0.10
Age	-0.02	0.01	0.142		-0.04	0.01
Race: Black[b]	-0.03	0.08	0.742		-0.18	0.13
Race: Hispanic[b]	-0.16	0.10	0.120		-0.35	0.04
Race: Other[b,c]	0.06	0.10	0.583		-0.15	0.26
Parent Education: High School[d]	0.12	0.06	0.053		0.00	0.24
Parent Education: Some College[d]	0.01	0.05	0.781		-0.08	0.11
Gender: Female[e]	-0.37	0.04	0.000	***	-0.45	-0.29
Proportion of Households under Poverty	0.10	0.20	0.609		-0.29	0.50
No. of Household Members	0.01	0.02	0.487		-0.03	0.05
Caregiver Smokes: Yes[f]	-0.07	0.06	0.219		-0.19	0.04
Epigenetic Assay: Batch 1[g]	0.33	0.24	0.169		-0.14	0.79
Epigenetic Assay: Batch 2[g]	0.39	0.23	0.090		-0.06	0.85
Time Since Loss	0.01	0.01	0.345		-0.01	0.02
**PhenoAge**						
	b	se	p		95% CI (Lower Limit)	95% CI (Upper Limit)
Parental Loss in Childhood: Yes[a]	-0.18	0.15	0.249		-0.48	0.12
Parental Loss in Adulthood: Yes[a]	0.03	0.07	0.661		-0.10	0.16
Age	-0.01	0.01	0.454		-0.03	0.02
Race: Black[b]	-0.20	0.07	0.006	**	-0.34	-0.06
Race: Hispanic[b]	-0.04	0.10	0.687		-0.25	0.16
Race: Other[b,c]	-0.05	0.13	0.705		-0.32	0.22
Parent Education: High School[d]	0.27	0.05	0.000	***	0.16	0.38
Parent Education: Some College[d]	0.17	0.06	0.006	**	0.05	0.29
Gender: Female[e]	0.10	0.04	0.011	*	0.02	0.18
Proportion of Households under Poverty	0.18	0.24	0.461		-0.29	0.64
No. of Household Members	-0.01	0.02	0.621		-0.05	0.03
Caregiver Smokes: Yes[f]	0.08	0.05	0.126		-0.02	0.18
Epigenetic Assay: Batch 1[g]	-0.15	0.15	0.333		-0.45	0.15
Epigenetic Assay: Batch 2[g]	-0.16	0.16	0.314		-0.47	0.15
Time Since Loss	0.01	0.01	0.051		0.00	0.02
**GrimAge**						

© 2024 Aiello AE et al. *JAMA Network Open*.

	b	se	p		95% CI (Lower Limit)	95% CI (Upper Limit)
Parental Loss in Childhood: Yes[a]	0.03	0.13	0.809		-0.22	0.28
Parental Loss in Adulthood: Yes[a]	0.15	0.06	0.011	*	0.04	0.27
Age	-0.05	0.01	0.000	***	-0.08	-0.03
Race: Black[b]	0.13	0.06	0.053	.	0.00	0.25
Race: Hispanic[b]	-0.41	0.09	0.000	***	-0.59	-0.23
Race: Other[b,c]	-0.06	0.10	0.513		-0.25	0.13
Parent Education: High School[d]	0.42	0.06	0.000	***	0.30	0.55
Parent Education: Some College[d]	0.29	0.05	0.000	***	0.19	0.40
Gender: Female[e]	-0.39	0.04	0.000	***	-0.47	-0.30
Proportion of Households under Poverty	0.47	0.25	0.062		-0.02	0.96
No. of Household Members	0.02	0.02	0.255		-0.02	0.07
Caregiver Smokes: Yes[f]	0.44	0.06	0.000	***	0.33	0.55
Epigenetic Assay: Batch 1[g]	-0.17	0.28	0.536		-0.72	0.38
Epigenetic Assay: Batch 2[g]	0.20	0.27	0.466		-0.34	0.74
Time Since Loss	0.00	0.00	0.377		-0.01	0.01

DunedinPACE

	b	se	p		95% CI (Lower Limit)	95% CI (Upper Limit)
Parental Loss in Childhood: Yes[a]	0.00	0.14	0.989		-0.27	0.27
Parental Loss in Adulthood: Yes[a]	0.15	0.06	0.025	*	0.02	0.28
Age	0.02	0.01	0.151		-0.01	0.04
Race: Black[b]	0.36	0.06	0.000	***	0.23	0.48
Race: Hispanic[b]	0.07	0.11	0.541		-0.15	0.29
Race: Other[b,c]	0.25	0.12	0.032	*	0.02	0.49
Parent Education: High School[d]	0.52	0.06	0.000	***	0.41	0.63
Parent Education: Some College[d]	0.30	0.05	0.000	***	0.19	0.40
Gender: Female[e]	0.17	0.04	0.000	***	0.09	0.26
Proportion of Households under Poverty	0.64	0.23	0.006	**	0.18	1.09
No. of Household Members	0.02	0.02	0.356		-0.02	0.06
Caregiver Smokes: Yes[f]	0.19	0.06	0.001	**	0.07	0.30
Epigenetic Assay: Batch 1[g]	0.06	0.12	0.656		-0.19	0.30
Epigenetic Assay: Batch 2[g]	0.13	0.12	0.270		-0.10	0.37
Time Since Loss	0.00	0.01	0.435		-0.01	0.01

Note: a = No Loss (reference) b = White (reference); c = Asian, Native American, Pacific Islander, Other (other race or multi-racial); d = College or Higher reference; e = Male (reference); f = No (reference); g = Batch 3 (reference)

*$p \leq 0.05$; **$p \leq 0.01$; ***$p \leq 0.001$

© 2024 Aiello AE et al. *JAMA Network Open*.

eTable 9. Number of Losses and Biological Aging (n = 3,963)

Horvath						
	b	se	p		95% CI (Lower Limit)	95% CI (Upper Limit)
One Loss: Yes[a]	-0.01	0.05	0.863		-0.10	0.08
Two or More Losses: Yes[a]	-0.08	0.07	0.252		-0.23	0.06
Age	-0.01	0.01	0.216		-0.04	0.01
Race: Black[b]	-0.02	0.08	0.753		-0.18	0.13
Race: Hispanic[b]	-0.16	0.10	0.098		-0.36	0.03
Race: Other[b, c]	0.09	0.09	0.274		-0.07	0.26
Parent Education: High School[d]	0.12	0.06	0.044	*	0.00	0.25
Parent Education: Some College[d]	0.03	0.05	0.597		-0.07	0.12
Gender: Female[e]	-0.37	0.04	0.000	***	-0.45	-0.28
Proportion of Households under Poverty	0.11	0.20	0.588		-0.28	0.50
No. of Household Members	0.02	0.02	0.435		-0.02	0.06
Caregiver Smokes: Yes[f]	-0.07	0.06	0.223		-0.19	0.04
Epigenetic Assay: Batch 1[g]	0.34	0.23	0.147		-0.12	0.80
Epigenetic Assay: Batch 2[g]	0.41	0.23	0.078		-0.05	0.86
PhenoAge						
	b	se	p		95% CI (Lower Limit)	95% CI (Upper Limit)
One Loss: Yes[a]	0.09	0.05	0.099		-0.02	0.19
Two or More Losses: Yes[a]	0.15	0.07	0.028	*	0.02	0.28
Age	-0.01	0.01	0.493		-0.03	0.02
Race: Black[b]	-0.19	0.07	0.010	*	-0.33	-0.05
Race: Hispanic[b]	-0.05	0.10	0.637		-0.24	0.15
Race: Other[b, c]	-0.01	0.13	0.957		-0.27	0.25
Parent Education: High School[d]	0.26	0.05	0.000	***	0.16	0.37
Parent Education: Some College[d]	0.16	0.06	0.009	**	0.04	0.28
Gender: Female[e]	0.10	0.04	0.011	*	0.02	0.18
Proportion of Households under Poverty	0.14	0.24	0.562		-0.33	0.60
No. of Household Members	-0.01	0.02	0.565		-0.06	0.03
Caregiver Smokes: Yes[f]	0.08	0.05	0.114		-0.02	0.18
Epigenetic Assay: Batch 1[g]	-0.14	0.15	0.345		-0.44	0.16
Epigenetic Assay: Batch 2[g]	-0.15	0.16	0.334		-0.46	0.16
GrimAge						
	b	se	p		95% CI (Lower Limit)	95% CI (Upper Limit)

	b	se	p		95% CI (Lower Limit)	95% CI (Upper Limit)
One Loss: Yes[a]	0.19	0.05	0.000	***	0.09	0.29
Two or More Losses: Yes[a]	0.27	0.09	0.003	**	0.09	0.45
Age	-0.05	0.01	0.000	***	-0.07	-0.03
Race: Black[b]	0.12	0.07	0.080		-0.01	0.24
Race: Hispanic[b]	-0.42	0.09	0.000	***	-0.59	-0.24
Race: Other[b, c]	-0.02	0.10	0.840		-0.23	0.18
Parent Education: High School[d]	0.41	0.06	0.000	***	0.29	0.53
Parent Education: Some College[d]	0.29	0.05	0.000	***	0.18	0.39
Gender: Female[e]	-0.40	0.04	0.000	***	-0.48	-0.31
Proportion of Households under Poverty	0.47	0.24	0.056		-0.01	0.95
No. of Household Members	0.02	0.02	0.336		-0.02	0.06
Caregiver Smokes: Yes[f]	0.46	0.06	0.000	***	0.34	0.57
Epigenetic Assay: Batch 1[g]	-0.15	0.29	0.597		-0.72	0.41
Epigenetic Assay: Batch 2[g]	0.21	0.28	0.446		-0.34	0.76
DunedinPACE						
	b	se	p		95% CI (Lower Limit)	95% CI (Upper Limit)
One Loss: Yes[a]	0.17	0.05	0.000	***	0.08	0.27
Two or More Losses: Yes[a]	0.22	0.06	0.000	***	0.10	0.34
Age	0.02	0.01	0.126		0.00	0.04
Race: Black[b]	0.35	0.07	0.000	***	0.22	0.47
Race: Hispanic[b]	0.06	0.10	0.540		-0.14	0.27
Race: Other[b, c]	0.28	0.11	0.016	*	0.05	0.50
Parent Education: High School[d]	0.50	0.05	0.000	***	0.39	0.61
Parent Education: Some College[d]	0.29	0.05	0.000	***	0.19	0.40
Gender: Female[e]	0.17	0.04	0.000	***	0.09	0.25
Proportion of Households under Poverty	0.64	0.23	0.006	**	0.18	1.09
No. of Household Members	0.02	0.02	0.378		-0.02	0.05
Caregiver Smokes: Yes[f]	0.19	0.06	0.001	**	0.08	0.31
Epigenetic Assay: Batch 1[g]	0.07	0.12	0.588		-0.18	0.31
Epigenetic Assay: Batch 2[g]	0.14	0.12	0.234		-0.09	0.38

Note: a = No Loss (reference) b = White (reference); c = Asian, Native American, Pacific Islander, Other (other race or multi-racial); d = College or Higher reference; e = Male (reference); f = No (reference); g = Batch 3 (reference)

*$p \leq 0.05$; **$p \leq 0.01$; ***$p \leq 0.001$

© 2024 Aiello AE et al. *JAMA Network Open*.

eTable 10. Parental Loss at Any Time Period (Childhood to Adulthood) and Biological Aging (*n* = 3,963)

	b	se	p		95% CI (Lower Limit)	95% CI (Upper Limit)
**Horvath**						
Parental Loss: Yes[a]	0.00	0.05	0.982		-0.09	0.09
Age	-0.02	0.01	0.177		-0.04	0.01
Race: Black[b]	-0.03	0.08	0.684		-0.18	0.12
Race: Hispanic[b]	-0.16	0.10	0.108		-0.35	0.04
Race: Other[b,c]	0.09	0.08	0.271		-0.07	0.26
Parent Education: High School[d]	0.12	0.06	0.055		0.00	0.24
Parent Education: Some College[d]	0.02	0.05	0.637		-0.07	0.12
Gender: Female[e]	-0.37	0.04	0.000	***	-0.45	-0.28
Proportion of Households under Poverty	0.09	0.20	0.647		-0.30	0.48
No. of Household Members	0.02	0.02	0.427		-0.02	0.06
Caregiver Smokes: Yes[f]	-0.08	0.06	0.198		-0.19	0.04
Epigenetic Assay: Batch 1[g]	0.33	0.23	0.156		-0.13	0.80
Epigenetic Assay: Batch 2[g]	0.40	0.23	0.083		-0.05	0.86
**PhenoAge**						
	b	se	p		95% CI (Lower Limit)	95% CI (Upper Limit)
Parental Loss: Yes[a]	0.12	0.05	0.014	*	0.02	0.21
Age	-0.01	0.01	0.505		-0.03	0.02
Race: Black[b]	-0.19	0.07	0.011	*	-0.33	-0.04
Race: Hispanic[b]	-0.05	0.10	0.630		-0.25	0.15
Race: Other[b,c]	-0.01	0.13	0.944		-0.27	0.25
Parent Education: High School[3]	0.27	0.05	0.000	***	0.16	0.38
Parent Education: Some College[3]	0.16	0.06	0.008	**	0.04	0.28
Gender: Female[4]	0.10	0.04	0.011	*	0.02	0.18
Proportion of Households under Poverty	0.14	0.24	0.546		-0.33	0.61
No. of Household Members	-0.01	0.02	0.625		-0.05	0.03
Caregiver Smokes: Yes[f]	0.08	0.05	0.118		-0.02	0.19
Epigenetic Assay: Batch 1[g]	-0.15	0.15	0.328		-0.45	0.15
Epigenetic Assay: Batch 2[g]	-0.16	0.16	0.312		-0.47	0.15
**GrimAge**						
	b	se	p		95% CI (Lower Limit)	95% CI (Upper Limit)
Parental Loss: Yes[a]	0.21	0.05	0.000	***	0.12	0.31

© 2024 Aiello AE et al. *JAMA Network Open*.

	b	se	p		95% CI (Lower Limit)	95% CI (Upper Limit)
Age	-0.05	0.01	0.000	***	-0.07	-0.03
Race: Black[b]	0.12	0.06	0.061		-0.01	0.25
Race: Hispanic[b]	-0.42	0.09	0.000	***	-0.60	-0.24
Race: Other[b,c]	-0.02	0.10	0.836		-0.23	0.18
Parent Education: High School[d]	0.42	0.06	0.000	***	0.30	0.54
Parent Education: Some College[d]	0.29	0.05	0.000	***	0.19	0.40
Gender: Female[e]	-0.40	0.04	0.000	***	-0.48	-0.31
Proportion of Households under Poverty	0.48	0.25	0.053		-0.01	0.97
No. of Household Members	0.02	0.02	0.278		-0.02	0.07
Caregiver Smokes: Yes[f]	0.46	0.06	0.000	***	0.34	0.57
Epigenetic Assay: Batch 1[g]	-0.16	0.28	0.573		-0.72	0.40
Epigenetic Assay: Batch 2[g]	0.20	0.28	0.466		-0.35	0.75
DunedinPACE						
	b	se	p		95% CI (Lower Limit)	95% CI (Upper Limit)
Parental Loss: Yes[a]	0.19	0.04	0.000	***	0.11	0.28
Age	0.02	0.01	0.117		0.00	0.04
Race: Black[b]	0.35	0.07	0.000	***	0.22	0.48
Race: Hispanic[b]	0.07	0.11	0.548		-0.15	0.28
Race: Other[b,c]	0.27	0.11	0.017	*	0.05	0.50
Parent Education: High School[d]	0.51	0.06	0.000	***	0.40	0.62
Parent Education: Some College[d]	0.29	0.05	0.000	***	0.19	0.40
Gender: Female[e]	0.17	0.04	0.000	***	0.09	0.25
Proportion of Households under Poverty	0.64	0.23	0.006	**	0.19	1.10
No. of Household Members	0.02	0.02	0.313		-0.02	0.06
Caregiver Smokes: Yes[f]	0.19	0.06	0.001	**	0.08	0.31
Epigenetic Assay: Batch 1[g]	0.06	0.12	0.647		-0.19	0.30
Epigenetic Assay: Batch 2[g]	0.13	0.12	0.273		-0.10	0.37

Note: a = No Loss (reference) b = White (reference); c = Asian, Native American, Pacific Islander, Other (other race or multi-racial); d = College or Higher reference; e = Male (reference); f = No (reference); g = Batch 3 (reference)

*$p \leq 0.05$; **$p \leq 0.01$; ***$p \leq 0.001$

© 2024 Aiello AE et al. *JAMA Network Open*.

eTable 11. Any Loss in Childhood and in Adulthood and Biological Aging (n = 3,963)						
Horvath						
	b	se	p		95% CI (Lower Limit)	95% CI (Upper Limit)
Any Loss in Childhood: Yes[a]	-0.07	0.08	0.371		-0.22	0.08
Any Loss in Adulthood: Yes[a]	-0.01	0.05	0.883		-0.10	0.09
Age	-0.02	0.01	0.178		-0.04	0.01
Race: Black[b]	-0.03	0.08	0.730		-0.18	0.13
Race: Hispanic[b]	-0.16	0.10	0.107		-0.35	0.03
Race: Other[b,c]	0.10	0.09	0.266		-0.07	0.26
Parent Education: High School[d]	0.12	0.06	0.049	*	0.00	0.25
Parent Education: Some College[d]	0.02	0.05	0.645		-0.07	0.12
Gender: Female[e]	-0.36	0.04	0.000	***	-0.45	-0.28
Proportion of Households under Poverty	0.10	0.19	0.616		-0.29	0.48
No. of Household Members	0.02	0.02	0.445		-0.02	0.06
Caregiver Smokes: Yes[f]	-0.07	0.06	0.215		-0.19	0.04
Epigenetic Assay: Batch 1[g]	0.34	0.23	0.155		-0.13	0.80
Epigenetic Assay: Batch 2[g]	0.40	0.23	0.083		-0.05	0.86
PhenoAge						
	b	se	p		95% CI (Lower Limit)	95% CI (Upper Limit)
Any Loss in Childhood: Yes[a]	0.07	0.08	0.398		-0.09	0.23
Any Loss in Adulthood: Yes[a]	0.08	0.05	0.081		-0.01	0.18
Age	-0.01	0.01	0.535		-0.03	0.02
Race: Black[b]	-0.18	0.07	0.013	*	-0.32	-0.04
Race: Hispanic[b]	-0.05	0.10	0.647		-0.25	0.16
Race: Other[b,c]	0.00	0.13	0.979		-0.27	0.26
Parent Education: High School[d]	0.27	0.05	0.000	***	0.16	0.38
Parent Education: Some College[d]	0.16	0.06	0.008	**	0.04	0.28
Gender: Female[e]	0.10	0.04	0.012	*	0.02	0.18
Proportion of Households under Poverty	0.14	0.24	0.549		-0.33	0.61
No. of Household Members	-0.01	0.02	0.582		-0.05	0.03
Caregiver Smokes: Yes[f]	0.08	0.05	0.109		-0.02	0.19
Epigenetic Assay: Batch 1[g]	-0.14	0.15	0.363		-0.44	0.16
Epigenetic Assay: Batch 2[g]	-0.15	0.16	0.344		-0.46	0.16
GrimAge						
	b	se	p		95% CI (Lower Limit)	95% CI (Upper Limit)

© 2024 Aiello AE et al. *JAMA Network Open*.

	b	se	p		95% CI (Lower Limit)	95% CI (Upper Limit)
Any Loss in Childhood: Yes[a]	0.11	0.09	0.207		-0.06	0.28
Any Loss in Adulthood: Yes[a]	0.16	0.05	0.001	**	0.07	0.26
Age	-0.05	0.01	0.000	***	-0.07	-0.03
Race: Black[b]	0.13	0.07	0.048	*	0.00	0.26
Race: Hispanic[b]	-0.41	0.09	0.000	***	-0.59	-0.24
Race: Other[b,c]	-0.01	0.11	0.920		-0.22	0.20
Parent Education: High School[d]	0.42	0.06	0.000	***	0.30	0.54
Parent Education: Some College[d]	0.29	0.05	0.000	***	0.19	0.40
Gender: Female[e]	-0.40	0.04	0.000	***	-0.48	-0.32
Proportion of Households under Poverty	0.48	0.24	0.053		-0.01	0.96
No. of Household Members	0.02	0.02	0.324		-0.02	0.06
Caregiver Smokes: Yes[f]	0.46	0.06	0.000	***	0.34	0.58
Epigenetic Assay: Batch 1[g]	-0.14	0.29	0.622		-0.71	0.43
Epigenetic Assay: Batch 2[g]	0.22	0.28	0.433		-0.33	0.77
DunedinPACE						
	b	se	p		95% CI (Lower Limit)	95% CI (Upper Limit)
Any Loss in Childhood: Yes[a]	0.08	0.08	0.344		-0.08	0.24
Any Loss in Adulthood: Yes[a]	0.15	0.05	0.002	**	0.06	0.24
Age	0.02	0.01	0.108		0.00	0.04
Race: Black[b]	0.36	0.06	0.000	***	0.23	0.49
Race: Hispanic[b]	0.07	0.11	0.516		-0.14	0.28
Race: Other[b,c]	0.29	0.12	0.015	*	0.06	0.51
Parent Education: High School[d]	0.51	0.05	0.000	***	0.41	0.62
Parent Education: Some College[d]	0.30	0.05	0.000	***	0.19	0.40
Gender: Female[e]	0.17	0.04	0.000	***	0.09	0.25
Proportion of Households under Poverty	0.64	0.23	0.006	**	0.19	1.10
No. of Household Members	0.02	0.02	0.373		-0.02	0.05
Caregiver Smokes: Yes[f]	0.20	0.06	0.001	***	0.08	0.31
Epigenetic Assay: Batch 1[g]	0.07	0.13	0.555		-0.17	0.32
Epigenetic Assay: Batch 2[g]	0.15	0.12	0.220		-0.09	0.38

Note: a = No Loss (reference) b = White (reference); c = Asian, Native American, Pacific Islander, Other (other race or multi-racial); d = College or Higher reference; e = Male (reference); f = No (reference); g = Batch 3 (reference)

*$p \leq 0.05$; **$p \leq 0.01$; ***$p \leq 0.001$

© 2024 Aiello AE et al. *JAMA Network Open*.

eTable 12. Parental Loss in Childhood and in Adulthood and Biological Aging (*n* = 3,963)

Horvath						
	b	se	p		95% CI (Lower Limit)	95% CI (Upper Limit)
Parental Loss in Childhood: Yes[a]	-0.07	0.08	0.357		-0.23	0.09
Parental Loss in Adulthood: Yes[a]	0.02	0.05	0.627		-0.07	0.12
Age	-0.02	0.01	0.160		-0.04	0.01
Race: Black[b]	-0.03	0.08	0.691		-0.18	0.12
Race: Hispanic[b]	-0.16	0.10	0.110		-0.35	0.04
Race: Other[b,c]	0.09	0.08	0.265		-0.07	0.26
Parent Education: High School[d]	0.12	0.06	0.052		0.00	0.24
Parent Education: Some College[d]	0.02	0.05	0.664		-0.08	0.12
Gender: Female[e]	-0.36	0.04	0.000	***	-0.45	-0.28
Proportion of Households under Poverty	0.09	0.20	0.658		-0.30	0.47
No. of Household Members	0.02	0.02	0.432		-0.02	0.06
Caregiver Smokes: Yes[f]	-0.08	0.06	0.192		-0.19	0.04
Epigenetic Assay: Batch 1[g]	0.33	0.23	0.158		-0.13	0.79
Epigenetic Assay: Batch 2[g]	0.40	0.23	0.085		-0.06	0.85
PhenoAge						
	b	se	p		95% CI (Lower Limit)	95% CI (Upper Limit)
Parental Loss in Childhood: Yes[a]	0.07	0.08	0.393		-0.09	0.24
Parental Loss in Adulthood: Yes[a]	0.10	0.05	0.035	*	0.01	0.20
Age	-0.01	0.01	0.516		-0.03	0.02
Race: Black[b]	-0.18	0.07	0.013	*	-0.32	-0.04
Race: Hispanic[b]	-0.05	0.10	0.639		-0.25	0.15
Race: Other[b,c]	-0.01	0.13	0.969		-0.27	0.26
Parent Education: High School[d]	0.27	0.05	0.000	***	0.16	0.38
Parent Education: Some College[d]	0.16	0.06	0.008	**	0.04	0.28
Gender: Female[e]	0.10	0.04	0.011	*	0.02	0.18
Proportion of Households under Poverty	0.15	0.24	0.536		-0.32	0.62
No. of Household Members	-0.01	0.02	0.610		-0.05	0.03
Caregiver Smokes: Yes[f]	0.08	0.05	0.115		-0.02	0.19
Epigenetic Assay: Batch 1[g]	-0.15	0.15	0.339		-0.45	0.16
Epigenetic Assay: Batch 2[g]	-0.16	0.16	0.321		-0.46	0.15
GrimAge						
	b	se	p		95% CI (Lower Limit)	95% CI (Upper Limit)

© 2024 Aiello AE et al. *JAMA Network Open*.

	b	se	p		95% CI (Lower Limit)	95% CI (Upper Limit)
Parental Loss in Childhood: Yes[a]	0.11	0.10	0.260		-0.08	0.31
Parental Loss in Adulthood: Yes[a]	0.17	0.05	0.001	**	0.07	0.27
Age	-0.05	0.01	0.000	***	-0.07	-0.03
Race: Black[b]	0.13	0.07	0.045	*	0.00	0.26
Race: Hispanic[b]	-0.41	0.09	0.000	***	-0.59	-0.24
Race: Other[b,c]	-0.01	0.11	0.906		-0.22	0.20
Parent Education: High School[d]	0.42	0.06	0.000	***	0.30	0.54
Parent Education: Some College[d]	0.30	0.05	0.000	***	0.19	0.40
Gender: Female[e]	-0.40	0.04	0.000	***	-0.48	-0.31
Proportion of Households under Poverty	0.49	0.25	0.048	*	0.00	0.98
No. of Household Members	0.02	0.02	0.311		-0.02	0.06
Caregiver Smokes: Yes[f]	0.46	0.06	0.000	***	0.35	0.58
Epigenetic Assay: Batch 1[g]	-0.15	0.29	0.603		-0.72	0.42
Epigenetic Assay: Batch 2[g]	0.21	0.28	0.445		-0.34	0.76
DunedinPACE						
	b	se	p		95% CI (Lower Limit)	95% CI (Upper Limit)
Parental Loss in Childhood: Yes[a]	0.09	0.09	0.337		-0.09	0.26
Parental Loss in Adulthood: Yes[a]	0.17	0.05	0.000	***	0.08	0.26
Age	0.02	0.01	0.111		0.00	0.04
Race: Black[b]	0.36	0.06	0.000	***	0.23	0.49
Race: Hispanic[b]	0.07	0.11	0.531		-0.15	0.28
Race: Other[b,c]	0.28	0.11	0.015	*	0.06	0.51
Parent Education: High School[d]	0.51	0.06	0.000	***	0.40	0.62
Parent Education: Some College[d]	0.30	0.05	0.000	***	0.19	0.40
Gender: Female[e]	0.17	0.04	0.000	***	0.09	0.25
Proportion of Households under Poverty	0.65	0.23	0.005	**	0.20	1.10
No. of Household Members	0.02	0.02	0.348		-0.02	0.05
Caregiver Smokes: Yes[f]	0.20	0.06	0.001	***	0.08	0.31
Epigenetic Assay: Batch 1[g]	0.07	0.12	0.600		-0.18	0.31
Epigenetic Assay: Batch 2[g]	0.14	0.12	0.246		-0.10	0.37

Note: a = No Loss (reference) b = White (reference); c = Asian, Native American, Pacific Islander, Other (other race or multi-racial); d = College or Higher reference; e = Male (reference); f = No (reference); g = Batch 3 (reference)

*$p \leq 0.05$; **$p \leq 0.01$; ***$p \leq 0.001$

© 2024 Aiello AE et al. *JAMA Network Open*.

eTable 13. Number of Losses and Biological Aging (*n* = 3,362)

Horvath

	b	se	p		95% CI (Lower Limit)	95% CI (Upper Limit)
One Loss: Yes[a]	-0.03	0.05	0.593		-0.13	0.08
Two or More Losses: Yes[a]	-0.07	0.10	0.478		-0.27	0.13
Age	-0.01	0.01	0.291		-0.04	0.01
Race: Black[b]	-0.03	0.10	0.775		-0.22	0.17
Parent Education: High School[c]	0.10	0.07	0.135		-0.03	0.23
Parent Education: Some College[c]	-0.02	0.05	0.761		-0.11	0.08
Gender: Female[d]	-0.36	0.05	0.000	***	-0.45	-0.27
Proportion of Households under Poverty	0.26	0.20	0.182		-0.13	0.65
No. of Household Members	0.03	0.02	0.149		-0.01	0.07
Caregiver Smokes: Yes[e]	-0.10	0.06	0.076		-0.21	0.01
Epigenetic Assay: Batch 1[f]	0.32	0.28	0.247		-0.23	0.87
Epigenetic Assay: Batch 2[f]	0.38	0.27	0.168		-0.16	0.92
One Loss* Race	0.00	0.14	0.993		-0.27	0.27
Two or More Losses*Race	-0.09	0.18	0.630		-0.43	0.26

PhenoAge

	b	se	p		95% CI (Lower Limit)	95% CI (Upper Limit)
One Loss: Yes[a]	0.09	0.05	0.083		-0.01	0.20
Two or More Losses: Yes[a]	0.06	0.09	0.509		-0.12	0.24
Age	0.00	0.01	0.914		-0.03	0.03
Race: Black[b]	-0.17	0.10	0.100		-0.37	0.03
Parent Education: High School[c]	0.27	0.06	0.000	***	0.15	0.39
Parent Education: Some College[c]	0.15	0.06	0.014	*	0.03	0.28
Gender: Female[d]	0.09	0.04	0.033	*	0.01	0.18
Proportion of Households under Poverty	0.19	0.25	0.444		-0.30	0.68
No. of Household Members	-0.01	0.02	0.654		-0.06	0.03
Caregiver Smokes: Yes[e]	0.06	0.05	0.232		-0.04	0.17
Epigenetic Assay: Batch 1[f]	-0.24	0.15	0.117		-0.55	0.06
Epigenetic Assay: Batch 2[f]	-0.26	0.16	0.102		-0.57	0.05
One Loss* Race	-0.15	0.15	0.341		-0.45	0.16
Two or More Losses*Race	0.16	0.18	0.356		-0.19	0.52

GrimAge

© 2024 Aiello AE et al. *JAMA Network Open*.

	b	se	p		95% CI (Lower Limit)	95% CI (Upper Limit)
One Loss: Yes[a]	0.23	0.06	0.000	***	0.12	0.35
Two or More Losses: Yes[a]	0.22	0.12	0.075		-0.02	0.47
Age	-0.05	0.01	0.000	***	-0.07	-0.02
Race: Black[b]	0.12	0.08	0.166		-0.05	0.28
Parent Education: High School[c]	0.44	0.07	0.000	***	0.31	0.57
Parent Education: Some College[c]	0.30	0.06	0.000	***	0.19	0.42
Gender: Female[d]	-0.41	0.05	0.000	***	-0.51	-0.31
Proportion of Households under Poverty	0.60	0.28	0.033	*	0.05	1.15
No. of Household Members	0.03	0.02	0.232		-0.02	0.08
Caregiver Smokes: Yes[e]	0.44	0.06	0.000	***	0.33	0.56
Epigenetic Assay: Batch 1[f]	-0.20	0.31	0.525		-0.82	0.42
Epigenetic Assay: Batch 2[f]	0.15	0.31	0.628		-0.46	0.76
One Loss* Race	-0.14	0.13	0.291		-0.40	0.12
Two or More Losses*Race	0.12	0.19	0.545		-0.27	0.50

DunedinPACE

	b	se	p		95% CI (Lower Limit)	95% CI (Upper Limit)
One Loss: Yes[a]	0.21	0.05	0.00	***	0.11	0.32
Two or More Losses: Yes[a]	0.12	0.09	0.18		-0.06	0.29
Age	0.02	0.01	0.03	*	0.00	0.05
Race: Black[b]	0.35	0.09	0.00	***	0.17	0.53
Parent Education: High School[c]	0.51	0.06	0.00	***	0.40	0.63
Parent Education: Some College[c]	0.31	0.05	0.00	***	0.20	0.42
Gender: Female[d]	0.16	0.04	0.00	***	0.08	0.25
Proportion of Households under Poverty	0.58	0.24	0.02	*	0.11	1.05
No. of Household Members	0.02	0.02	0.25		-0.02	0.06
Caregiver Smokes: Yes[e]	0.19	0.06	0.00	**	0.07	0.30
Epigenetic Assay: Batch 1[f]	0.15	0.15	0.32		-0.14	0.43
Epigenetic Assay: Batch 2[f]	0.22	0.14	0.13		-0.06	0.50
One Loss* Race	-0.14	0.14	0.31		-0.42	0.14
Two or More Losses*Race	0.29	0.17	0.08	.	-0.04	0.62

Note: a = No Loss (reference) b = White (reference); c = College or Higher reference; d = Male (reference); e = No (reference); f = Batch 3 (reference)

*$p \leq 0.05$; **$p \leq 0.01$; ***$p \leq 0.001$

© 2024 Aiello AE et al. *JAMA Network Open*.

eTable 14. Parental Loss at Any Time Period (Childhood to Adulthood) and Biological Aging (*n* = 3,362)

Horvath						
	b	se	p		95% CI (Lower Limit)	95% CI (Upper Limit)
Parental Loss: Yes[a]	-0.02	0.05	0.730		-0.12	0.08
Age	-0.01	0.01	0.246		-0.04	0.01
Race: Black[b]	-0.07	0.10	0.459		-0.26	0.12
Parent Education: High School[c]	0.10	0.07	0.159		-0.04	0.23
Parent Education: Some College[c]	-0.02	0.05	0.754		-0.11	0.08
Gender: Female[d]	-0.36	0.05	0.000	***	-0.46	-0.27
Proportion of Households under Poverty	0.23	0.19	0.235		-0.15	0.62
No. of Household Members	0.03	0.02	0.155		-0.01	0.07
Caregiver Smokes: Yes[e]	-0.10	0.06	0.064		-0.22	0.01
Epigenetic Assay: Batch 1[f]	0.32	0.28	0.252		-0.23	0.86
Epigenetic Assay: Batch 2[f]	0.37	0.27	0.170		-0.16	0.91
Parental Loss*Race	0.05	0.12	0.690		-0.19	0.28
PhenoAge						
	b	se	p		95% CI (Lower Limit)	95% CI (Upper Limit)
Parental Loss: Yes[a]	0.10	0.06	0.084		-0.01	0.21
Age	0.00	0.01	0.957		-0.03	0.03
Race: Black[b]	-0.23	0.10	0.019	*	-0.42	-0.04
Parent Education: High School[c]	0.27	0.06	0.000	***	0.15	0.39
Parent Education: Some College[c]	0.16	0.06	0.012	*	0.04	0.28
Gender: Female[d]	0.10	0.04	0.021	*	0.02	0.19
Proportion of Households under Poverty	0.19	0.25	0.450		-0.31	0.69
No. of Household Members	-0.01	0.02	0.762		-0.05	0.04
Caregiver Smokes: Yes[e]	0.05	0.05	0.304		-0.05	0.16
Epigenetic Assay: Batch 1[f]	-0.26	0.15	0.096		-0.56	0.05
Epigenetic Assay: Batch 2[f]	-0.27	0.16	0.080		-0.58	0.03
Parental Loss*Race	0.08	0.13	0.545		-0.18	0.34
GrimAge						
	b	se	p		95% CI (Lower Limit)	95% CI (Upper Limit)
Parental Loss: Yes[a]	0.23	0.06	0.000	***	0.11	0.35
Age	-0.05	0.01	0.001	***	-0.07	-0.02
Race: Black[b]	0.10	0.08	0.186		-0.05	0.26

© 2024 Aiello AE et al. *JAMA Network Open*.

	b	se	p		95% CI (Lower Limit)	95% CI (Upper Limit)
Parent Education: High School[c]	0.46	0.07	0.000	***	0.33	0.59
Parent Education: Some College[c]	0.31	0.06	0.000	***	0.19	0.43
Gender: Female[d]	-0.40	0.05	0.000	***	-0.50	-0.31
Proportion of Households under Poverty	0.62	0.28	0.031	*	0.06	1.18
No. of Household Members	0.03	0.02	0.176		-0.02	0.08
Caregiver Smokes: Yes[e]	0.44	0.06	0.000	***	0.32	0.55
Epigenetic Assay: Batch 1[f]	-0.21	0.31	0.497		-0.83	0.40
Epigenetic Assay: Batch 2[f]	0.13	0.30	0.658		-0.47	0.74
Parental Loss*Race	-0.03	0.12	0.826		-0.26	0.21

DunedinPACE

	b	se	p		95% CI (Lower Limit)	95% CI (Upper Limit)
Parental Loss: Yes[a]	0.18	0.05	0.000	***	0.09	0.28
Age	0.03	0.01	0.023	*	0.00	0.05
Race: Black[b]	0.29	0.09	0.001	**	0.11	0.46
Parent Education: High School[c]	0.52	0.06	0.000	***	0.41	0.64
Parent Education: Some College[c]	0.32	0.05	0.000	***	0.21	0.42
Gender: Female[d]	0.18	0.04	0.000	***	0.09	0.26
Proportion of Households under Poverty	0.59	0.24	0.016	*	0.11	1.06
No. of Household Members	0.03	0.02	0.167		-0.01	0.07
Caregiver Smokes: Yes[e]	0.17	0.06	0.003	**	0.06	0.29
Epigenetic Assay: Batch 1[f]	0.13	0.14	0.372		-0.15	0.41
Epigenetic Assay: Batch 2[f]	0.20	0.14	0.163		-0.08	0.47
Parental Loss*Race	0.14	0.11	0.232		-0.09	0.36

Note: a = No Loss (reference) b = White (reference); c = College or Higher reference; d = Male (reference); e = No (reference); f = Batch 3 (reference)

*$p \leq 0.05$; **$p \leq 0.01$; ***$p \leq 0.001$

© 2024 Aiello AE et al. *JAMA Network Open*.

eTable 15. Any Loss in Childhood and in Adulthood and Biological Aging (*n* = 3,362)

	b	se	p		95% CI (Lower Limit)	95% CI (Upper Limit)
**Horvath**						
Any Loss in Childhood: Yes[a]	0.04	0.10	0.651		-0.15	0.24
Any Loss in Adulthood: Yes[a]	-0.03	0.06	0.575		-0.14	0.08
Age	-0.01	0.01	0.247		-0.04	0.01
Race: Black[b]	-0.04	0.10	0.689		-0.23	0.15
Parent Education: High School[c]	0.10	0.07	0.151		-0.04	0.23
Parent Education: Some College[c]	-0.02	0.05	0.759		-0.11	0.08
Gender: Female[d]	-0.36	0.05	0.000	***	-0.45	-0.27
Proportion of Households under Poverty	0.25	0.20	0.204		-0.14	0.64
No. of Household Members	0.03	0.02	0.143		-0.01	0.07
Caregiver Smokes: Yes[e]	-0.11	0.06	0.056		-0.22	0.00
Epigenetic Assay: Batch 1[f]	0.31	0.28	0.259		-0.23	0.86
Epigenetic Assay: Batch 2[f]	0.37	0.27	0.177		-0.17	0.90
Any Loss in Childhood* Race	-0.29	0.17	0.100		-0.63	0.06
Any Loss in Adulthood*Race	0.07	0.14	0.619		-0.20	0.34
**PhenoAge**						
	b	se	p		95% CI (Lower Limit)	95% CI (Upper Limit)
Any Loss in Childhood: Yes[a]	0.04	0.10	0.694		-0.16	0.24
Any Loss in Adulthood: Yes[a]	0.08	0.06	0.169		-0.03	0.19
Age	0.00	0.01	0.980		-0.03	0.03
Race: Black[b]	-0.21	0.10	0.037	*	-0.40	-0.01
Parent Education: High School[c]	0.27	0.06	0.000	***	0.15	0.39
Parent Education: Some College[c]	0.16	0.06	0.013	*	0.03	0.28
Gender: Female[d]	0.10	0.04	0.024	*	0.01	0.18
Proportion of Households under Poverty	0.20	0.25	0.436		-0.30	0.69
No. of Household Members	-0.01	0.02	0.719		-0.05	0.04
Caregiver Smokes: Yes[e]	0.06	0.05	0.273		-0.05	0.16
Epigenetic Assay: Batch 1[f]	-0.24	0.15	0.114		-0.55	0.06
Epigenetic Assay: Batch 2[f]	-0.26	0.16	0.096		-0.57	0.05
Any Loss in Childhood* Race	0.09	0.20	0.662		-0.30	0.48
Any Loss in Adulthood*Race	0.01	0.14	0.940		-0.26	0.28
**GrimAge**						
	b	se	p		95% CI (Lower Limit)	95% CI (Upper Limit)

© 2024 Aiello AE et al. *JAMA Network Open*.

Any Loss in Childhood: Yes[a]	0.09	0.11	0.430		-0.13	0.30
Any Loss in Adulthood: Yes[a]	0.19	0.06	0.002	**	0.07	0.30
Age	-0.05	0.01	0.001	***	-0.07	-0.02
Race: Black[b]	0.10	0.09	0.247		-0.07	0.28
Parent Education: High School[c]	0.46	0.07	0.000	***	0.33	0.59
Parent Education: Some College[c]	0.31	0.06	0.000	***	0.19	0.43
Gender: Female[d]	-0.41	0.05	0.000	***	-0.50	-0.31
Proportion of Households under Poverty	0.61	0.28	0.030	*	0.06	1.16
No. of Household Members	0.03	0.02	0.208		-0.02	0.08
Caregiver Smokes: Yes[e]	0.44	0.06	0.000	***	0.32	0.56
Epigenetic Assay: Batch 1[f]	-0.19	0.31	0.546		-0.81	0.43
Epigenetic Assay: Batch 2[f]	0.16	0.31	0.610		-0.45	0.76
Any Loss in Childhood* Race	0.14	0.20	0.487		-0.25	0.52
Any Loss in Adulthood*Race	-0.05	0.14	0.691		-0.32	0.22

DunedinPACE

	b	se	p		95% CI (Lower Limit)	95% CI (Upper Limit)
Any Loss in Childhood: Yes[a]	0.05	0.11	0.622		-0.16	0.27
Any Loss in Adulthood: Yes[a]	0.16	0.05	0.002	**	0.06	0.26
Age	0.03	0.01	0.021	*	0.00	0.05
Race: Black[b]	0.33	0.09	0.000	***	0.15	0.50
Parent Education: High School[c]	0.52	0.06	0.000	***	0.40	0.64
Parent Education: Some College[c]	0.31	0.05	0.000	***	0.21	0.42
Gender: Female[d]	0.17	0.04	0.000	***	0.08	0.26
Proportion of Households under Poverty	0.59	0.24	0.015	*	0.12	1.07
No. of Household Members	0.03	0.02	0.200		-0.01	0.07
Caregiver Smokes: Yes[e]	0.18	0.06	0.002	**	0.07	0.29
Epigenetic Assay: Batch 1[f]	0.15	0.15	0.310		-0.14	0.43
Epigenetic Assay: Batch 2[f]	0.22	0.14	0.129		-0.06	0.50
Any Loss in Childhood* Race	0.17	0.17	0.299		-0.16	0.50
Any Loss in Adulthood*Race	0.02	0.13	0.855		-0.23	0.28

Note: a = No Loss (reference) b = White (reference); c = College or Higher reference; d = Male (reference); e = No (reference); f = Batch 3 (reference)

*$p \leq 0.05$; **$p \leq 0.01$; ***$p \leq 0.001$

© 2024 Aiello AE et al. *JAMA Network Open*.

eTable 16. Parental Loss in Childhood and in Adulthood and Biological Aging (n = 3,362)

Horvath						
	b	se	p		95% CI (Lower Limit)	95% CI (Upper Limit)
Parental Loss in Childhood: Yes[a]	-0.02	0.11	0.855		-0.23	0.19
Parental Loss in Adulthood: Yes[a]	-0.01	0.06	0.915		-0.12	0.11
Age	-0.01	0.01	0.228		-0.04	0.01
Race: Black[b]	-0.09	0.09	0.370		-0.27	0.10
Parent Education: High School[c]	0.10	0.07	0.152		-0.04	0.23
Parent Education: Some College[c]	-0.02	0.05	0.762		-0.11	0.08
Gender: Female[d]	-0.36	0.05	0.000	***	-0.45	-0.27
Proportion of Households under Poverty	0.23	0.20	0.251		-0.16	0.61
No. of Household Members	0.03	0.02	0.148		-0.01	0.07
Caregiver Smokes: Yes[e]	-0.11	0.06	0.053		-0.22	0.00
Epigenetic Assay: Batch 1[f]	0.32	0.27	0.247		-0.22	0.86
Epigenetic Assay: Batch 2[f]	0.37	0.27	0.170		-0.16	0.90
Parental Loss in Childhood* Race	-0.13	0.19	0.487		-0.51	0.25
Parental Loss in Adulthood*Race	0.13	0.14	0.354		-0.15	0.41
**PhenoAge**						
	b	se	p		95% CI (Lower Limit)	95% CI (Upper Limit)
Parental Loss in Childhood: Yes[a]	0.01	0.11	0.902		-0.21	0.23
Parental Loss in Adulthood: Yes[a]	0.11	0.06	0.070	.	-0.01	0.22
Age	0.00	0.01	0.937		-0.03	0.03
Race: Black[b]	-0.22	0.09	0.016	*	-0.41	-0.04
Parent Education: High School[c]	0.27	0.06	0.000	***	0.15	0.39
Parent Education: Some College[c]	0.16	0.06	0.013	*	0.03	0.28
Gender: Female[d]	0.10	0.04	0.023	*	0.01	0.18
Proportion of Households under Poverty	0.20	0.25	0.437		-0.30	0.70
No. of Household Members	-0.01	0.02	0.741		-0.05	0.04
Caregiver Smokes: Yes[e]	0.06	0.05	0.273		-0.05	0.16
Epigenetic Assay: Batch 1[f]	-0.26	0.15	0.095	.	-0.56	0.05
Epigenetic Assay: Batch 2[f]	-0.27	0.16	0.081	.	-0.58	0.03
Parental Loss in Childhood* Race	0.25	0.19	0.205		-0.14	0.63
Parental Loss in Adulthood*Race	0.02	0.14	0.859		-0.25	0.30
**GrimAge**						
	b	se	p		95% CI (Lower Limit)	95% CI (Upper Limit)
Parental Loss in Childhood: Yes[a]	0.14	0.13	0.275		-0.11	0.39

Parental Loss in Adulthood: Yes[a]	0.19	0.06	0.002	**	0.07	0.30
Age	-0.05	0.01	0.001	***	-0.07	-0.02
Race: Black[b]	0.11	0.08	0.179		-0.05	0.27
Parent Education: High School[c]	0.46	0.07	0.000	***	0.33	0.59
Parent Education: Some College[c]	0.31	0.06	0.000	***	0.19	0.43
Gender: Female[d]	-0.40	0.05	0.000	***	-0.50	-0.31
Proportion of Households under Poverty	0.63	0.28	0.027	*	0.07	1.19
No. of Household Members	0.03	0.02	0.186		-0.02	0.08
Caregiver Smokes: Yes[e]	0.44	0.06	0.000	***	0.32	0.56
Epigenetic Assay: Batch 1[f]	-0.20	0.31	0.515		-0.82	0.41
Epigenetic Assay: Batch 2[f]	0.14	0.30	0.639		-0.46	0.75
Parental Loss in Childhood* Race	0.02	0.21	0.911		-0.40	0.44
Parental Loss in Adulthood*Race	-0.03	0.14	0.829		-0.30	0.24

DunedinPACE

	b	se	p		95% CI (Lower Limit)	95% CI (Upper Limit)
Parental Loss in Childhood: Yes[a]	0.09	0.12	0.427		-0.14	0.33
Parental Loss in Adulthood: Yes[a]	0.16	0.05	0.003	**	0.06	0.26
Age	0.03	0.01	0.021	*	0.00	0.05
Race: Black[b]	0.29	0.09	0.002	**	0.11	0.47
Parent Education: High School[c]	0.53	0.06	0.000	***	0.41	0.65
Parent Education: Some College[c]	0.32	0.05	0.000	***	0.21	0.42
Gender: Female[d]	0.17	0.04	0.000	***	0.09	0.26
Proportion of Households under Poverty	0.60	0.24	0.014	*	0.13	1.07
No. of Household Members	0.03	0.02	0.184		-0.01	0.07
Caregiver Smokes: Yes[e]	0.18	0.06	0.003	**	0.06	0.29
Epigenetic Assay: Batch 1[f]	0.13	0.14	0.349		-0.15	0.41
Epigenetic Assay: Batch 2[f]	0.20	0.14	0.148		-0.07	0.47
Parental Loss in Childhood* Race	0.17	0.19	0.367		-0.20	0.54
Parental Loss in Adulthood*Race	0.14	0.13	0.278		-0.11	0.39

Note: a = No Loss (reference) b = White (reference); c = College or Higher reference; d = Male (reference); e = No (reference); f = Batch 3 (reference)

*$p \leq 0.05$; **$p \leq 0.01$; ***$p \leq 0.001$

© 2024 Aiello AE et al. *JAMA Network Open*.

eTable 17. Parental Loss by Gender of Parent at Any Time Period (Childhood to Adulthood) and Biological Aging (*n* = 3,963)

Horvath						
	b	se	p		95% CI (Lower Limit)	95% CI (Upper Limit)
Parental Loss (Mother or Mother Figure): Yes[a]	0.00	0.07	0.98		-0.13	0.13
Parental Loss (Father or Father Figure): Yes[a]	-0.02	0.05	0.71		-0.11	0.08
Age	-0.02	0.01	0.14		-0.04	0.01
Race: Black[b]	-0.04	0.07	0.62		-0.18	0.11
Race: Hispanic[b]	-0.16	0.10	0.11		-0.36	0.03
Race: Other[b,c]	0.09	0.08	0.27		-0.07	0.26
Parent Education: High School[d]	0.13	0.06	0.04	*	0.01	0.25
Parent Education: Some College[d]	0.02	0.05	0.65		-0.08	0.12
Gender: Female[e]	-0.36	0.04	0.00	***	-0.45	-0.28
Proportion of Households under Poverty	0.06	0.20	0.75		-0.33	0.45
No. of Household Members	0.02	0.02	0.34		-0.02	0.06
Caregiver Smokes: Yes[f]	-0.08	0.06	0.18		-0.20	0.04
Epigenetic Assay: Batch 1[g]	0.33	0.24	0.16		-0.13	0.80
Epigenetic Assay: Batch 2[g]	0.40	0.23	0.08		-0.05	0.86

PhenoAge						
	b	se	p		95% CI (Lower Limit)	95% CI (Upper Limit)
Parental Loss (Mother or Mother Figure): Yes[a]	0.13	0.06	0.04	*	0.01	0.25
Parental Loss (Father or Father Figure): Yes[a]	0.09	0.05	0.08		-0.01	0.20
Age	-0.01	0.01	0.42		-0.04	0.01
Race: Black[b]	-0.20	0.07	0.01	**	-0.34	-0.05
Race: Hispanic[b]	-0.05	0.10	0.64		-0.25	0.16
Race: Other[b,c]	-0.01	0.13	0.96		-0.27	0.26
Parent Education: High School[d]	0.27	0.05	0.00	***	0.16	0.38
Parent Education: Some College[d]	0.16	0.06	0.01	**	0.04	0.28
Gender: Female[e]	0.11	0.04	0.01	**	0.03	0.19
Proportion of Households under Poverty	0.12	0.24	0.62		-0.36	0.60
No. of Household Members	-0.01	0.02	0.72		-0.05	0.03
Caregiver Smokes: Yes[f]	0.07	0.05	0.17		-0.03	0.18
Epigenetic Assay: Batch 1[g]	-0.15	0.15	0.31		-0.45	0.15
Epigenetic Assay: Batch 2[g]	-0.16	0.16	0.31		-0.47	0.15

GrimAge

© 2024 Aiello AE et al. *JAMA Network Open*.

	b	se	p		95% CI (Lower Limit)	95% CI (Upper Limit)
Parental Loss (Mother or Mother Figure): Yes[a]	0.16	0.07	0.02	*	0.02	0.30
Parental Loss (Father or Father Figure): Yes[a]	0.18	0.05	0.00	***	0.08	0.28
Age	-0.05	0.01	0.00	***	-0.07	-0.03
Race: Black[b]	0.13	0.07	0.05		0.00	0.26
Race: Hispanic[b]	-0.41	0.09	0.00	***	-0.59	-0.23
Race: Other[b,c]	-0.02	0.10	0.88		-0.22	0.19
Parent Education: High School[d]	0.41	0.06	0.00	***	0.29	0.53
Parent Education: Some College[d]	0.29	0.05	0.00	***	0.19	0.40
Gender: Female[e]	-0.40	0.04	0.00	***	-0.48	-0.31
Proportion of Households under Poverty	0.49	0.25	0.05	*	0.01	0.98
No. of Household Members	0.02	0.02	0.35		-0.02	0.06
Caregiver Smokes: Yes[f]	0.46	0.06	0.00	***	0.35	0.58
Epigenetic Assay: Batch 1[g]	-0.16	0.29	0.58		-0.73	0.41
Epigenetic Assay: Batch 2[g]	0.20	0.28	0.47		-0.35	0.75

DunedinPACE

	b	se	p		95% CI (Lower Limit)	95% CI (Upper Limit)
Parental Loss (Mother or Mother Figure): Yes[a]	0.21	0.06	0.00	**	0.08	0.34
Parental Loss (Father or Father Figure): Yes[a]	0.14	0.04	0.00	**	0.05	0.22
Age	0.02	0.01	0.12		0.00	0.04
Race: Black[b]	0.35	0.07	0.00	***	0.22	0.48
Race: Hispanic[b]	0.07	0.11	0.50		-0.14	0.29
Race: Other[b,c]	0.28	0.11	0.02	*	0.05	0.50
Parent Education: High School[d]	0.50	0.05	0.00	***	0.40	0.61
Parent Education: Some College[d]	0.30	0.05	0.00	***	0.19	0.40
Gender: Female[e]	0.17	0.04	0.00	***	0.09	0.25
Proportion of Households under Poverty	0.65	0.23	0.01	**	0.20	1.11
No. of Household Members	0.02	0.02	0.38		-0.02	0.05
Caregiver Smokes: Yes[f]	0.20	0.06	0.00	***	0.08	0.31
Epigenetic Assay: Batch 1[g]	0.06	0.13	0.61		-0.18	0.31
Epigenetic Assay: Batch 2[g]	0.13	0.12	0.26		-0.10	0.37

Note: a = No Loss (reference) b = White (reference); c = Asian, Native American, Pacific Islander, Other (other race or multi-racial); d = College or Higher reference; e = Male (reference); f = No (reference); g = Batch 3 (reference)

*$p \leq 0.05$; **$p \leq 0.01$; ***$p \leq 0.001$

© 2024 Aiello AE et al. *JAMA Network Open*.

eTable 18. Interaction Between Any Loss in Childhood and in Adulthood and its Association With Biological Aging (*n* = 3,963)

Horvath						
	b	se	p		95% CI (Lower Limit)	95% CI (Upper Limit)
Any Loss in Childhood: Yes[a]	-0.02	0.09	0.82		-0.20	0.16
Any Loss in Adulthood: Yes[a]	0.00	0.05	0.93		-0.10	0.10
Age	-0.02	0.01	0.19		-0.04	0.01
Race: Black[b]	-0.03	0.08	0.70		-0.18	0.12
Race: Hispanic[b]	-0.16	0.10	0.10		-0.35	0.03
Race: Other[b,c]	0.10	0.08	0.27		-0.07	0.26
Parent Education: High School[d]	0.12	0.06	0.05		0.00	0.25
Parent Education: Some College[d]	0.02	0.05	0.65		-0.08	0.12
Gender: Female[e]	-0.36	0.04	0.00	***	-0.45	-0.28
Proportion of Households under Poverty	0.10	0.19	0.59		-0.28	0.49
No. of Household Members	0.02	0.02	0.44		-0.02	0.06
Caregiver Smokes: Yes[f]	-0.07	0.06	0.22		-0.19	0.04
Epigenetic Assay: Batch 1[g]	0.33	0.23	0.16		-0.13	0.80
Epigenetic Assay: Batch 2[g]	0.40	0.23	0.08		-0.05	0.85
Any Loss in Childhood*Any Loss in Adulthood	-0.13	0.17	0.43		-0.46	0.20
**PhenoAge**						
	b	se	p		95% CI (Lower Limit)	95% CI (Upper Limit)
Any Loss in Childhood: Yes[a]	0.19	0.11	0.08		-0.03	0.40
Any Loss in Adulthood: Yes[a]	0.11	0.05	0.02	*	0.02	0.21
Age	-0.01	0.01	0.56		-0.03	0.02
Race: Black[b]	-0.19	0.07	0.01	**	-0.33	-0.05
Race: Hispanic[b]	-0.05	0.10	0.62		-0.25	0.15
Race: Other[b,c]	0.00	0.13	0.98		-0.27	0.26
Parent Education: High School[d]	0.27	0.05	0.00	***	0.16	0.38
Parent Education: Some College[d]	0.16	0.06	0.01	**	0.04	0.28
Gender: Female[e]	0.10	0.04	0.01	*	0.02	0.18
Proportion of Households under Poverty	0.16	0.24	0.51		-0.31	0.63
No. of Household Members	-0.01	0.02	0.60		-0.05	0.03
Caregiver Smokes: Yes[f]	0.09	0.05	0.09		-0.01	0.19
Epigenetic Assay: Batch 1[g]	-0.15	0.15	0.34		-0.45	0.16
Epigenetic Assay: Batch 2[g]	-0.16	0.16	0.33		-0.47	0.16
Any Loss in Childhood*Any Loss in Adulthood	-0.33	0.17	0.05		-0.66	0.00
**GrimAge**						

© 2024 Aiello AE et al. *JAMA Network Open*.

	b	se	p		95% CI (Lower Limit)	95% CI (Upper Limit)
Any Loss in Childhood: Yes[a]	0.17	0.11	0.12		-0.05	0.38
Any Loss in Adulthood: Yes[a]	0.18	0.05	0.00	***	0.08	0.28
Age	-0.05	0.01	0.00	***	-0.07	-0.03
Race: Black[b]	0.13	0.06	0.05		0.00	0.25
Race: Hispanic[b]	-0.41	0.09	0.00	***	-0.59	-0.24
Race: Other[b,c]	-0.01	0.11	0.92		-0.22	0.20
Parent Education: High School[d]	0.42	0.06	0.00	***	0.30	0.54
Parent Education: Some College[d]	0.29	0.05	0.00	***	0.19	0.40
Gender: Female[e]	-0.40	0.04	0.00	***	-0.48	-0.31
Proportion of Households under Poverty	0.49	0.24	0.05	*	0.00	0.97
No. of Household Members	0.02	0.02	0.32		-0.02	0.06
Caregiver Smokes: Yes[f]	0.46	0.06	0.00	***	0.34	0.58
Epigenetic Assay: Batch 1[g]	-0.15	0.29	0.61		-0.72	0.43
Epigenetic Assay: Batch 2[g]	0.22	0.28	0.44		-0.34	0.77
Any Loss in Childhood*Any Loss in Adulthood	-0.16	0.18	0.36		-0.51	0.19

DunedinPACE

	b	se	p		95% CI (Lower Limit)	95% CI (Upper Limit)
Any Loss in Childhood: Yes[a]	0.20	0.10	0.06		0.00	0.40
Any Loss in Adulthood: Yes[a]	0.18	0.05	0.00	***	0.09	0.27
Age	0.02	0.01	0.10		0.00	0.04
Race: Black[b]	0.35	0.07	0.00	***	0.22	0.48
Race: Hispanic[b]	0.07	0.11	0.54		-0.15	0.28
Race: Other[b,c]	0.29	0.12	0.01	*	0.06	0.51
Parent Education: High School[d]	0.51	0.06	0.00	***	0.40	0.62
Parent Education: Some College[d]	0.30	0.05	0.00	***	0.19	0.40
Gender: Female[e]	0.17	0.04	0.00	***	0.09	0.25
Proportion of Households under Poverty	0.66	0.23	0.00	**	0.20	1.11
No. of Household Members	0.02	0.02	0.36		-0.02	0.05
Caregiver Smokes: Yes[f]	0.20	0.06	0.00	***	0.09	0.32
Epigenetic Assay: Batch 1[g]	0.07	0.13	0.59		-0.18	0.32
Epigenetic Assay: Batch 2[g]	0.14	0.12	0.25		-0.10	0.38
Any Loss in Childhood*Any Loss in Adulthood	-0.34	0.15	0.03	*	-0.64	-0.04

Note: a = No Loss (reference) b = White (reference); c = Asian, Native American, Pacific Islander, Other (other race or multi-racial); d = College or Higher reference; e = Male (reference); f = No (reference); g = Batch 3 (reference)

$*p \leq 0.05$; $**p \leq 0.01$; $***p \leq 0.001$

© 2024 Aiello AE et al. *JAMA Network Open*.

eTable 19. Interaction Between Parental Loss in Childhood and in Adulthood and its Association With Biological Aging ([illegible] 3,963)

	b	se	p		95% CI (Lower Limit)	95%[illegible] (Upper[illegible]
**Horvath**						
Parental Loss in Childhood: Yes[a]	-0.10	0.09	0.30		-0.29	[illegible]
Parental Loss in Adulthood: Yes[a]	0.02	0.05	0.72		-0.08	[illegible]
Age	-0.02	0.01	0.16		-0.04	[illegible]
Race: Black[b]	-0.03	0.08	0.71		-0.18	[illegible]
Race: Hispanic[b]	-0.16	0.10	0.11		-0.35	[illegible]
Race: Other[b,c]	0.09	0.08	0.27		-0.07	[illegible]
Parent Education: High School[d]	0.12	0.06	0.05	.	0.00	[illegible]
Parent Education: Some College[d]	0.02	0.05	0.66		-0.08	[illegible]
Gender: Female[e]	-0.36	0.04	0.00	***	-0.45	[illegible]
Proportion of Households under Poverty	0.09	0.20	0.66		-0.30	[illegible]
No. of Household Members	0.02	0.02	0.43		-0.02	[illegible]
Caregiver Smokes: Yes[f]	-0.08	0.06	0.19		-0.19	[illegible]
Epigenetic Assay: Batch 1[g]	0.33	0.23	0.16		-0.13	[illegible]
Epigenetic Assay: Batch 2[g]	0.40	0.23	0.09	.	-0.06	[illegible]
Parental Loss in Childhood*Parental Loss in Adulthood	0.09	0.20	0.67		-0.32	[illegible]
**PhenoAge**						
	b	se	p		95% CI (Lower Limit)	95%[illegible] (Upper[illegible]
Parental Loss in Childhood: Yes[a]	0.12	0.11	0.26		-0.09	[illegible]
Parental Loss in Adulthood: Yes[a]	0.12	0.05	0.02	*	0.02	[illegible]
Age	-0.01	0.01	0.52		-0.03	[illegible]
Race: Black[b]	-0.19	0.07	0.01	**	-0.33	[illegible]
Race: Hispanic[b]	-0.05	0.10	0.64		-0.25	[illegible]
Race: Other[b,c]	0.00	0.13	0.98		-0.27	[illegible]
Parent Education: High School[d]	0.27	0.05	0.00	***	0.16	[illegible]
Parent Education: Some College[d]	0.16	0.06	0.01	**	0.04	[illegible]
Gender: Female[e]	0.10	0.04	0.01	*	0.02	[illegible]
Proportion of Households under Poverty	0.15	0.24	0.53		-0.32	[illegible]
No. of Household Members	-0.01	0.02	0.61		-0.05	[illegible]
Caregiver Smokes: Yes[f]	0.09	0.05	0.11		-0.02	[illegible]
Epigenetic Assay: Batch 1[g]	-0.15	0.15	0.34		-0.45	[illegible]
Epigenetic Assay: Batch 2[g]	-0.16	0.16	0.32		-0.47	[illegible]
Parental Loss in Childhood*Parental Loss in Adulthood	-0.19	0.19	0.33		-0.57	[illegible]
**GrimAge**						

© 2024 Aiello AE et al. *JAMA Network Open*.

	b	se	p		95% CI (Lower Limit)	95% C (Upper L
Parental Loss in Childhood: Yes[a]	0.17	0.12	0.14		-0.06	0.41
Parental Loss in Adulthood: Yes[a]	0.18	0.05	0.00	***	0.08	0.28
Age	-0.05	0.01	0.00	***	-0.07	-0.03
Race: Black[b]	0.13	0.06	0.05	.	0.00	0.25
Race: Hispanic[b]	-0.42	0.09	0.00	***	-0.59	-0.24
Race: Other[b,c]	-0.01	0.11	0.92		-0.22	0.20
Parent Education: High School[d]	0.42	0.06	0.00	***	0.30	0.54
Parent Education: Some College[d]	0.29	0.05	0.00	***	0.19	0.40
Gender: Female[e]	-0.40	0.04	0.00	***	-0.48	-0.31
Proportion of Households under Poverty	0.49	0.25	0.05	*	0.01	0.98
No. of Household Members	0.02	0.02	0.31		-0.02	0.06
Caregiver Smokes: Yes[f]	0.46	0.06	0.00	***	0.35	0.58
Epigenetic Assay: Batch 1[g]	-0.15	0.29	0.60		-0.72	0.42
Epigenetic Assay: Batch 2[g]	0.21	0.28	0.45		-0.34	0.76
Parental Loss in Childhood*Parental Loss in Adulthood	-0.24	0.19	0.23		-0.62	0.15

DunedinPACE

	b	se	p		95% CI (Lower Limit)	95% C (Upper L
Parental Loss in Childhood: Yes[a]	0.19	0.11	0.08	.	-0.02	0.41
Parental Loss in Adulthood: Yes[a]	0.19	0.05	0.00	***	0.10	0.28
Age	0.02	0.01	0.10		0.00	0.04
Race: Black[b]	0.35	0.07	0.00	***	0.22	0.48
Race: Hispanic[b]	0.07	0.11	0.54		-0.15	0.28
Race: Other[b,c]	0.29	0.11	0.01	*	0.06	0.51
Parent Education: High School[d]	0.51	0.06	0.00	***	0.40	0.62
Parent Education: Some College[d]	0.30	0.05	0.00	***	0.19	0.40
Gender: Female[e]	0.17	0.04	0.00	***	0.09	0.25
Proportion of Households under Poverty	0.65	0.23	0.01	**	0.20	1.11
No. of Household Members	0.02	0.02	0.34		-0.02	0.05
Caregiver Smokes: Yes[f]	0.20	0.06	0.00	***	0.09	0.32
Epigenetic Assay: Batch 1[g]	0.06	0.12	0.61		-0.18	0.31
Epigenetic Assay: Batch 2[g]	0.14	0.12	0.26		-0.10	0.37
Parental Loss in Childhood*Parental Loss in Adulthood	-0.40	0.19	0.04	*	-0.77	-0.02

Note: a = No Loss (reference) b = White (reference); c = Asian, Native American, Pacific Islander, Other (other race or m racial); d = College or Higher reference; e = Male (reference); f = No (reference); g = Batch 3 (reference)

*$p \leq 0.05$; **$p \leq 0.01$; ***$p \leq 0.001$

© 2024 Aiello AE et al. *JAMA Network Open*.
