# Supplementary material for: Familial Loss of a Loved One and Biological Aging: NIMHD Social Epigenomics Program
Source: JAMA Netw Open. 2024 Jul 29;7(7):e2421869. doi: 10.1001/jamanetworkopen.2024.21869 (PMC11287397; doi:10.1001/jamanetworkopen.2024.21869)
Supplement: Supplement 2. — Data Sharing Statement [file jamanetwopen-e2421869-s002.pdf]

Data Sharing Statement

Aiello. Familial Loss of a Loved One and Biological Aging. *JAMA Netw Open*. Published July 29, 2024. doi:10.1001/jamanetworkopen.2024.21869

Data
Data available: Yes
Data types: Deidentified participant data
How to access data: https://addhealth.cpc.unc.edu
When available: beginning date: 01-01-2025

Supporting Documents
Document types: None

Additional Information
Who can access the data: researchers whose proposed use of the data has been approved
Types of analyses: for a specified purpose
Mechanisms of data availability: with a signed data access agreement)
